# Plasma-Enhancement of Oxygen Pumping: The Role of Atomic Oxygen

**DOI:** 10.1021/acs.jpcc.6c02560

**Published:** 2026-08-24

**Authors:** Xingyu Chen, Aleksandr Pikalev, Susanta Bera, Mihalis N. Tsampas, Guan-Jun Zhang, M. C. M. van de Sanden

**Affiliations:** † School of Electrical Engineering, 12480 Xi’an Jiaotong University, Xi’an, 710049 Shaanxi, China; ‡ 55956 Dutch Institute for Fundamental Energy Research (DIFFER), De Zaale 20, 5612 AJ Eindhoven, The Netherlands; § Eindhoven Institute for Renewable Energy Systems (EIRES), 3169 Eindhoven University of Technology, 5612 AZ Eindhoven, The Netherlands

## Abstract

Sluggish surface oxygen reduction kinetics remain a principal barrier to efficient oxygen pumping in solid oxide electrochemical cells (SOECs), where dissociative adsorption of O_2_ on lanthanum strontium manganite (LSM) electrodes is widely established as the rate-limiting step, particularly under reduced oxygen partial pressure and cathodic polarization conditions. While low-temperature plasmas generate reactive oxygen species, their coupling to electrochemical oxygen transport has not been quantitatively addressed. This work couples an oxygen-containing glow discharge to a symmetric LSM|YSZ|LSM SOEC to directly supply reactive oxygen species to the SOEC cathode. A 180% increase in oxygen pumping capacity is observed at 585 °C in 1 Torr He (80%)–O_2_ (20%) plasma at 470 W. Open-circuit voltage (OCV) measurements reveal a shift in electrochemical equilibrium consistent with increased surface oxygen activity. A surface reaction model identifies atomic oxygen as the dominant contributor to this enhancement. These findings demonstrate plasma–electrochemical coupling as a viable route to solid oxide oxygen separation at reduced operating temperatures.

## Graphical abstract

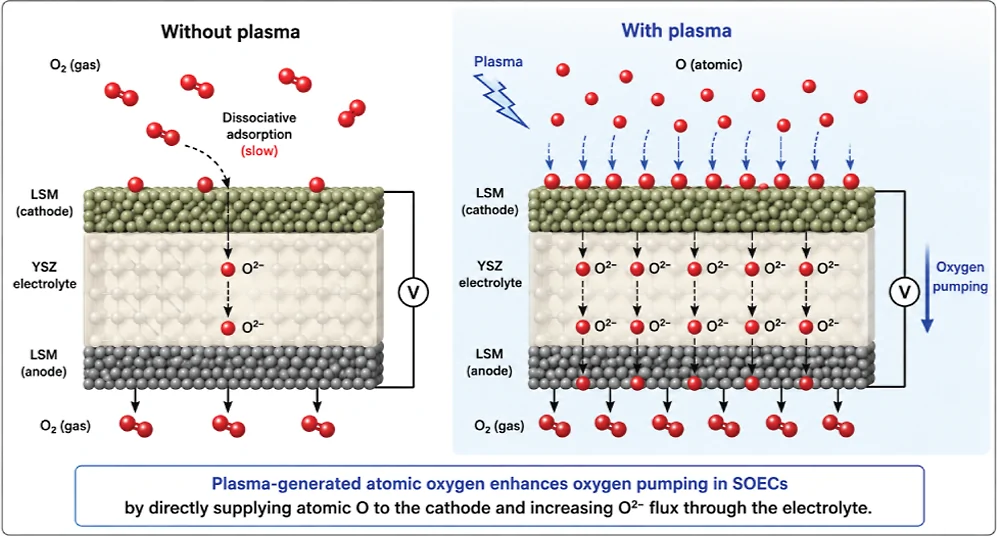

## Introduction

Efficient oxygen separation and transport underpin a wide range of energy and chemical processes, from gas stream separation and solid oxide fuel cells to CO_2_ conversion, where oxygen flux often determines overall system performance.^1–3^ Among current technologies, solid oxide electrochemical cells (SOECs) offer a thermodynamically efficient route for oxygen pumping at elevated temperatures, yet the performance is frequently limited by sluggish surface oxygen reduction kinetics at the cathodes.^4–6^

Low-temperature plasmas are well established as sources of reactive oxygen species,^7,8^ making them a compelling but largely unexplored tool for overcoming this kinetic barrier. Whether and how these plasma-generated oxygen species couple in electrochemical oxygen transport at solid oxide interfaces remains an open question. Specifically, the influence of plasma-driven oxygen fluxes on cathode equilibrium and interfacial oxygen reduction reaction (ORR) kinetics has not been quantitatively addressed.

The ORR (O_2_(g) + 4e^–^ → 2O^2–^) on LSM-based electrodes proceeds through sequential surface steps including adsorption, dissociation, charge transfer, and electrolyte incorporation, and is most severely rate-limited by dissociative adsorption under reduced oxygen partial pressure or cathodic polarization.^4,9–13^ Plasma activation generates atomic and excited oxygen species at densities far exceeding thermal equilibrium values and, provided these species reach the electrode surface before recombination, directly increases surface oxygen coverage and bypasses the dissociative adsorption barrier.^14–16^ The central open question is therefore whether plasma-induced oxygen radicals shift the electrochemical equilibrium at the SOEC cathode in a quantifiable and controllable way, or whether recombination and competing surface processes dissipate the plasma-induced flux before it contributes to oxygen transport.

Prior work has examined the coupling between low-temperature plasmas and oxide-ion conducting solid electrolytes from several aspects, though not in the context of SOEC-based oxygen pumping addressed here. Ash et al. showed that a DC glow discharge dissociating molecular oxygen upstream of a Ag 0.05Zr membrane increased oxygen permeation through the membrane by an order of magnitude at 450–550 °C, with an activation energy roughly half that of the unexcited case, and explicitly proposed the resulting atomic oxygen for direct delivery into a zirconia electrochemical pump; they attributed the enhancement to atomic oxygen bypassing the slow dissociative adsorption step that otherwise limits molecular O_2_ uptake on the metal surface.^17^ Rohnke et al. showed that exposing yttria-stabilized zirconia (YSZ) to a low-temperature oxygen radio frequency-plasma increases its ^18^O/^16^O surface exchange coefficient by up to 2 orders of magnitude relative to molecular oxygen, and discussed several possible mechanisms, including surface charging, UV irradiation, and atomic oxygen supply, without being able to conclusively isolate the atomic oxygen contribution.^18^ Steinmüller et al. subsequently demonstrated that replacing the metal electrodes of a low-pressure DC glow discharge with YSZ produces a functioning plasma–electrochemical cell in its own right, in which the ionic conductivity of the oxide electrode directly governs the discharge current–voltage behavior and optical emission, establishing YSZ as an active component of a plasma-coupled electrochemical system.^16^ At the reactor-integration level, Mori and Tun coupled a dielectric barrier discharge to a symmetric SOEC for CO_2_ decomposition, showing that continuous electrochemical removal of O_2_ by the SOEC suppressed the plasma-phase reverse reaction and allowed CO_2_ conversion to proceed toward completion rather than saturating, as it does in the plasma-only case. Taken together, these studies establish that plasma exposure measurably enhances oxygen exchange kinetics at oxide-ion conducting surfaces, that an oxide-ion conducting electrolyte can itself be integrated as an active element of a plasma–electrochemical system, and that hybridizing a discharge with an SOEC can shift plasma-driven gas-phase chemistry via electrochemical product removal.^19^ However, none of this prior work couples a well-characterized plasma directly and continuously to the working cathode of a closed, symmetric SOEC operating as an oxygen pump, nor develops a surface kinetic model that isolates the specific contribution of atomic oxygen to the resulting electrochemical performance. The present work addresses this gap: we interface a characterized He–O_2_ glow discharge directly with the LSM cathode of a symmetric SOEC under load, quantify the resulting shift in electrochemical equilibrium (OCV) and steady-state oxygen pumping flux, and develop a surface reaction model, validated against independently measured atomic-to-molecular oxygen flux ratios, that attributes the enhancement quantitatively to plasma-generated atomic oxygen.

Here, we directly probe the plasma–electrochemical coupling by interfacing an oxygen-containing glow discharge with a symmetric SOEC (LSM|YSZ|LSM). By varying plasma power and electrochemical conditions, we quantify how plasma-induced oxygen fluxes influence open cell voltage (OCV) and ORR kinetics. OCV shifts under plasma exposure are used as a direct thermodynamic probe of changes in surface oxygen activity, and a surface reaction model incorporating atomic oxygen fluxes is developed to separate plasma-driven contributions from intrinsic electrode behavior. The findings establish a quantitative framework for plasma–electrochemical coupling and point toward nonequilibrium plasma as a tool to enhance the oxygen separation and reduce the operating temperature of solid oxide oxygen pumping.^20,21^

## Results and Discussion

The temporal evolution of open-circuit voltage (OCV) under alternating plasma-on and plasma-off conditions in He–O_2_ plasma is shown in Figure 1. The OCV and current are measured directly between the two LSM electrodes of the SOEC under zero applied voltage; the SOEC floats electrically with respect to the plasma circuit throughout, as confirmed by an impedance-to-ground exceeding 10 MΩ. Before plasma ignition, the OCV maintains a stable baseline of −0.1275 (±4 × 10^–4^) V, consistent with the Nernst equilibrium potential^22^

1 $$E=\frac{RT}{4F}\;\ln\;\frac{pO_{2}^{cathode}}{pO_{2}^{anode}}$$

where *T* is the electrolyte temperature, *R* is the ideal gas constant and *F* is the Faraday constant. *p*O_2_^cathode^ and *p*O_2_^anode^ are the oxygen partial pressures at the cathode and anode, respectively.

**Figure 1:**
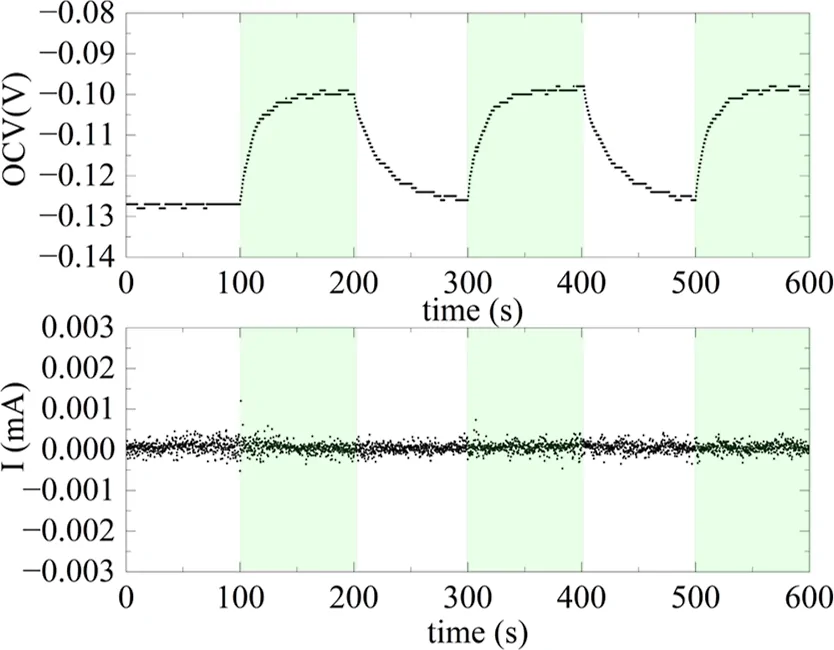
Temporal evolution of OCV measurements in 1 Torr He–O_2_ plasma, *p*O_2_ 0.2 Torr, *T*_SOEC_ 692 °C, *U*_plasma_ was set to −1100 V, *P*_plasma_ 471 W. Green regions are plasma on and white regions are without plasma.

Upon plasma ignition, the OCV shifts rapidly toward zero, reaching −0.0991 (±7 × 10^–4^) V with plasma on, which corresponds to an effective increase in cathode oxygen activity under plasma exposure. The response is fully reversible, with the OCV returning to baseline upon plasma extinction. The SOEC current remains within ±0.002 mA throughout, confirming that Faradaic oxygen transport is negligible and that the OCV shift reflects a change in surface thermodynamic equilibrium rather than a driven electrochemical process. To quantify the plasma effect on steady-state oxygen activity, we derive an equivalent oxygen partial pressure *p*O_2_^c,eq^ by inverting the Nernst equation

2 $$pO_{2}^{c,eq}=pO_{2}^{c,no\;plasma}\times e^{\left(\frac{4F}{RT}\cdot \Delta E\right)}$$

where Δ*E* = *E*_plasma on_ – *E*_no plasma_ is the plasma induced OCV shift and $pO_{2}^{c,no\;plasma}$ is the cathode oxygen partial pressure in the absence of plasma discharge. The steady-state OCV and corresponding $pO_{2}^{eq}$ as a function of plasma power are shown in Figure 2. Without plasma, the OCV stabilizes at −0.1275 (±4 × 10^–4^) V, corresponding to $pO_{2}^{eq}$ of ≈0.2 Torr baseline, consistent with the inlet composition and confirming Nernst equilibrium. At 215 W plasma power, the OCV shifts to −0.1180 (±2 × 10^–4^) V, yielding $pO_{2}^{eq}$ 0.32 Torr, a 60% increase from the baseline. At maximum tested power (471 W), the OCV reaches −0.0991 (±7 × 10^–4^) V and $pO_{2}^{eq}$ = 0.89 Torr, a 342% increase relative to the inlet *p*O_2_. The monotonic dependence of $pO_{2}^{eq}$ on plasma power indicates that atomic oxygen delivery to the cathode surface scales with discharge intensity, consistent with higher atomic oxygen production at elevated plasma power.

**Figure 2:**
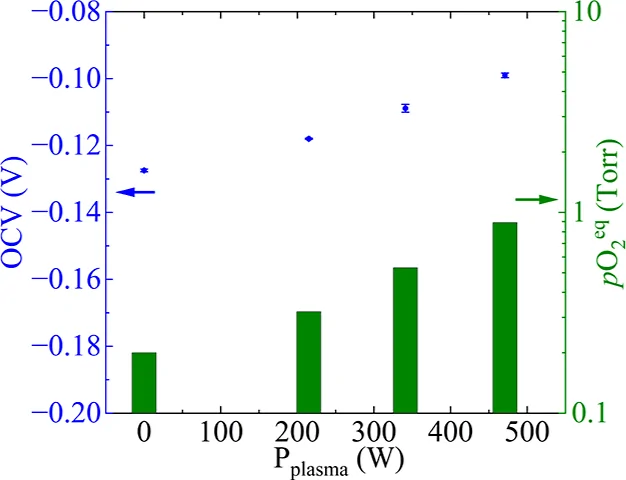
OCV steady response to He–O_2_ plasma, inlet *p*O_2_ 0.2 Torr, *T*_SOEC_ 692 °C, *U*_plasma_ was set to −900/–1000/–1100 V, corresponding *P*_plasma_ 215/341/471 W.

To connect the observed OCV response to plasma-surface chemistry, we examine how plasma-generated oxygen fluxes modify cathode surface kinetics. In the absence of plasma, oxygen reduction on the LSM cathode proceeds through the established sequential steps,^23^ as visualized in Figure 3a

3 $$O_{2}\left(g\right)\to 2O_{ad}$$

4 $$O_{ad}+e^{-}\to O_{ad}^{-}$$

5 $$O_{ad}^{-}\to O_{TPB}^{-}$$

6 $$O_{TPB}^{-}+e^{-}\to O_{YSZ}^{2-}$$

where “ad” denotes adsorbed surface species and “TPB” denotes the triple phase boundary, i.e., the contact line between the LSM electrode, YSZ electrolyte, and gas phase at which charge transfer and oxygen ion incorporation into the electrolyte occur.

**Figure 3:**
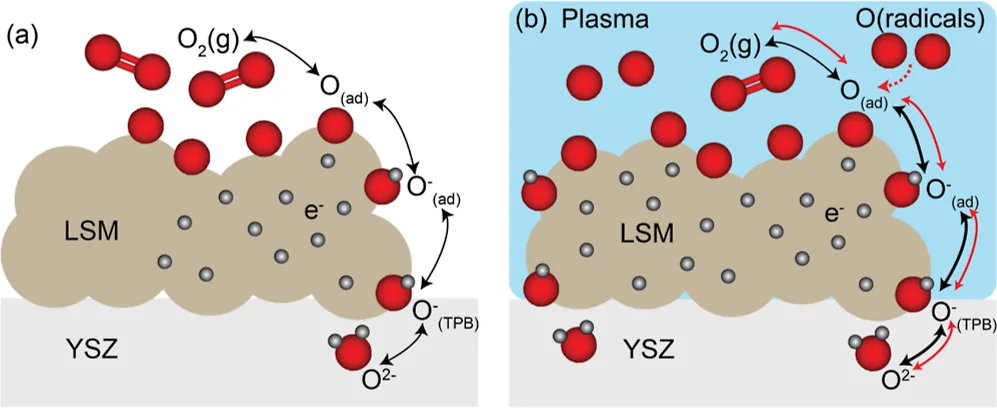
Visualization of oxygen reduction reactions at the LSM|YSZ interface: (a) thermal equilibrium and (b) plasma exposure. In panel (b), black arrows represent the conventional molecular oxygen reduction pathway operative under both thermal and plasma conditions, and red arrows indicate the adsorption pathway introduced by plasma-generated atomic oxygen species.

To relate plasma-induced OCV shifts to changes in surface oxygen coverage, we express the OCV via the generalized Nernst equation (see Supporting Information S2 for derivation)

7 $$E_{OCV}=\frac{RT}{4F}\;\ln\;\frac{pO_{2}\left(0\right)}{pO_{2}\left(L\right)}=\frac{RT}{2F}\;\ln\;\frac{n_{O_{ad}}\left(0\right)}{n_{O_{ad}}\left(L\right)}$$

where *p*O_2_(0) and *p*O_2_(*L*) are the oxygen partial pressure at the cathode and anode side, respectively. The prefactor changes from $\frac{RT}{4F}$ to $\frac{RT}{2F}$ because, under adsorption equilibrium, $pO_{2}\propto n_{O_{ad}}^{2}$ . The adsorbed oxygen surface density is defined as *n*_O_ad__ = *n*_0_^′^·θ_O_, where *n*_0_^′^ is the surface site density (m^–2^) and θ_O_ is the fractional surface coverage of oxygen atoms without the plasma (dimensionless). For the conditions studied here we neglect the influence of the ions since the flux of ions is much lower than the flux of atomic oxygen (Supporting Information, Figure S6).

Under He–O_2_ feed gas conditions, the surface oxygen coverage at the SOEC cathode evolves as

8 $$\begin{array}{l} \frac{dn_{O_{ad}}}{dt}=\Gamma_{ads}-\Gamma_{des}\\ =\Gamma_{O_{2}}\cdot \gamma_{O_{2}}-K_{des}\cdot n_{O_{ad}}^{2} \end{array}$$

where Γ_O_2__ is the molecular flux of O_2_ at the cathode surface (m^–2^·s^–1^), γ_O_2__ is the dissociative adsorption sticking coefficient for O_2_ molecules (dimensionless), and *K*_des_ is the second-order rate constant for associative desorption of adsorbed oxygen atoms to form O_2_ (m^2^·s^–1^). Equation 8 is applied under open-circuit conditions, where the net electrochemical current is zero and eq 4 is quasi-equilibrated: it carries no net flux and therefore constitutes no net sink for O_ad_, dropping out of the surface balance. At the resulting low equilibrium surface coverage (θ_O,eq_ ≪ 1, consistent with the *p*O_2_ power-law exponent of 0.263 ± 0.005 (see in Supporting Information, Figure S2) and with the low-coverage surface oxygen expected^23^), the associative recombination ( $K_{des}\cdot n_{O_{ad}}^{2}$ ) is the dominant loss channel for O_ad_. Therefore, the charge-transfer term is omitted from the surface balance. Under plasma exposure, additional flux contributions from atomic oxygen modify the surface coverage balance, as incorporated in the extended model described in Figure 3b.

Under plasma exposure, atomic oxygen delivered to the LSM cathode surface supplements the molecular O_2_ flux, modifying the adsorption–desorption balance and establishing a new surface kinetic equilibrium (Figure 3b)

9 $$\begin{array}{l} \frac{dn_{O_{ad}}}{dt}=\Gamma_{ads}^{plasma}-\Gamma_{des}^{plasma}\\ =\Gamma_{O_{2}}\gamma_{O_{2}}+\Gamma_{O}\gamma_{O}-K_{des}\cdot n_{O_{ad}}^{2} \end{array}$$

where Γ_O_ is the atomic oxygen flux from plasma dissociation (m^–2^·s^–1^), γ_O_ is the atomic oxygen sticking coefficient. *n*_O_ad__ = *n*_0_^′^·θ_Op_ is the number density of the adsorbed oxygen atoms (m^–2^), and θ_Op_ is the fractional surface coverage of oxygen atoms under He–O_2_ plasma conditions. Flux measurements in the plasma afterglow confirm that atomic oxygen constitutes the dominant reactive species reaching the cathode surface under the present experimental conditions (Supporting Information, Figure S6); the enhanced surface oxygen activity observed in He–O_2_ plasma is therefore attributed primarily to the Γ_O_γ_O_ term. In fact, this means that the assumed low surface coverage limit is valid.

When the SOEC cathode reaches equilibrium, there is no net change in surface oxygen concentration *n*_O_ad__, and in this steady state $\frac{dn_{O_{ad}}}{dt}=0$ . Analytical solutions to eqs 9 and 8 are fitted to the measured OCV transient response (see Supporting Information S3). The model reproduces the plasma-on and plasma-off OCV responses (Figure 4, with fitting parameters given in Supporting Information, Table S1). The fitted products are (Γ_O_2__γ_O_2__ + Γ_O_γ_O_)·*K*_des_ = (1.395 ± 0.0093) × 10^–3^ s^–2^ under plasma-on conditions and Γ_O_2__γ_O_2__·*K*_des_ = (4.8816 ± 0.0069) × 10^–4^ s^–2^ under plasma-off conditions. The OCV transient time scale reflects the intrinsic surface kinetics of oxygen adsorption–desorption on the porous LSM cathode rather than thermal or gas-phase transport effects. The thermal diffusion time across the YSZ wall (τ_thermal_ = ρ·*c*_*p*_·δ^2^/*k* ≈ 3 s) is an order of magnitude shorter than the observed transient, and the in situ YSZ conductivity thermometry confirms that the bulk electrolyte temperature remains stable within ±3.5 K during plasma cycling, a variation insufficient to account for the observed 30 mV OCV shift, which would require a temperature change of 200 K. The full reversibility and reproducibility of the OCV response across multiple on/off cycles (Figure 1) further rules out any cumulative thermal perturbation. Gas-phase diffusion equilibration is also far faster than the observed time scale. Instead, the characteristic equilibration time extracted from the surface reaction model fitting (τ = 1/|*k*_2_| ≈ 13–23 s, Table S1) is entirely consistent with the rate-limiting role of oxygen surface exchange on LSM at low *p*O_2_ in the mbar range, as established directly by Fleig et al.^11^ using LSM thin film microelectrodes on YSZ. Using the surface exchange coefficient *k* ^*Q*^ 4 × 10^–9^ cm/s from Fleig et al. and representative porous LSM electrode parameters, the characteristic surface exchange time τ = *L*/(*k* ^*Q*^·*a*) is of order 10–100 s, directly consistent with our observations.

**Figure 4:**
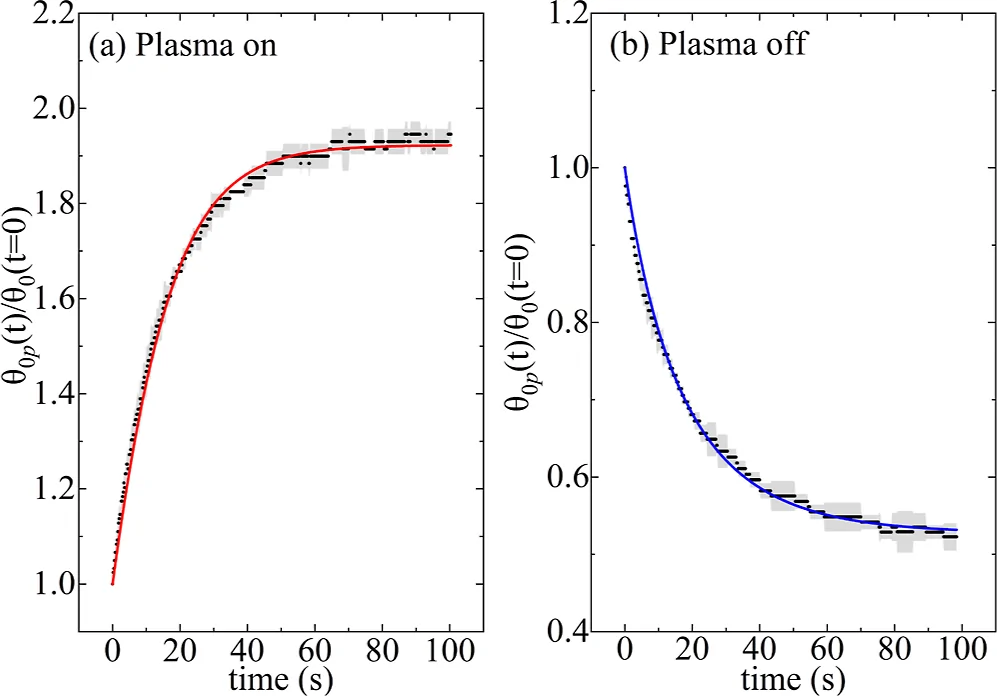
Surface coverage response to 1 Torr He–O_2_ plasma. Red and blue lines are the fitting curves for (a) plasma on and (b) plasma off. *U*_plasma_ −1100 V, *P*_plasma_ 471 W, *p*O_2_ 0.2 Torr, inlet flow 10 sccm, *T*_SOEC_ 692 °C. OCV data are averaged from measurements in Figure 1, with standard deviation indicated by shading.

These fitted products yield the atomic-to-molecular oxygen flux ratio at *U*_plasma_ −1100 V (*P*_plasma_ 471 W). Since *K*_des_ depends primarily on SOEC temperature and is independent of the ion and electron fluxes, which are 3 orders of magnitude lower than the atomic oxygen flux and 5 orders of magnitude lower than the molecular oxygen flux under these plasma conditions (Supporting Information, Figure S6), *K*_des_ cancels in the ratio

$$\begin{array}{l} \frac{\left(\Gamma_{O_{2}}\gamma_{O_{2}}+\Gamma_{O}\gamma_{O}\right)\cdot K_{des}}{\Gamma_{O_{2}}\gamma_{O_{2}}\cdot K_{des}}=\frac{\Gamma_{O_{2}}\gamma_{O_{2}}+\Gamma_{O}\gamma_{O}}{\Gamma_{O_{2}}\gamma_{O_{2}}}=2.86\pm 0.04\\ \frac{\Gamma_{O}\gamma_{O}}{\Gamma_{O_{2}}\gamma_{O_{2}}}\approx 1.86 \end{array}$$

Under our experimental conditions (low oxygen partial pressure *p*O_2_ < 0.001 atm, high surface temperature *T*_SOEC_ 692 °C), the sticking coefficient of atomic oxygen γ_O_ is significantly higher than that of molecular oxygen γ_O_2__, consistent with the dissociative adsorption barrier being bypassed for atomic species.^24^ Direct oxygen radical measurements (Supporting Information, Figure S6) give the ratio $\frac{\Gamma_{O}}{\Gamma_{O_{2}}}=\left(6.51\pm 2.50\right)\times 10^{-3}$ , from which we extract: $\frac{\gamma_{O}}{\gamma_{O_{2}}}$ = 286 (±110) under our experimental conditions. The large relative uncertainty in this ratio reflects the 38% uncertainty in the measured $\frac{\Gamma_{O}}{\Gamma_{O_{2}}}$ ratio. Nevertheless, the result is consistent with literature values of $\frac{\gamma_{O}}{\gamma_{O_{2}}}$ in the range of 100–400 at comparable temperatures.^25–27^ This result is reproduced at a different plasma condition (*U*_plasma_ −1000 V, *P*_plasma_ 341 W), as shown in the Supporting Information (Figure S4 and Table S2).

This differential sticking behavior between atomic and molecular oxygen on oxide cathodes is also the operating principle of YSZ-based amperometric atomic oxygen sensors, which use electrodes of different catalytic activity to discriminate atomic from molecular oxygen flux, since different materials support dissociative O_2_ adsorption to differing degrees. Examples include the FIPEX sensor family and its derivative APOLLON payload developed by Zajac et al. for satellite-based measurement,^28^ a class of device reviewed comprehensively by Holmes et al. alongside competing atomic-oxygen sensing technologies.^29^ More recently, Hoerner et al. demonstrated an improved thin-film FIPEX variant (eFIPEX) in which selectivity toward atomic oxygen is achieved specifically through gold plating of the platinum electrode surface, since gold suppresses dissociative adsorption of molecular O_2_ while remaining responsive to atomic oxygen already present in the gas phase, directly exploiting the same sticking probability contrast central to the surface reaction model developed here.^30^ These sensors confirm, at the level of device engineering, that the differential sticking probability of atomic versus molecular oxygen on solid-oxide and noble-metal-coated cathodes is a robust and exploitable effect. This prior work is, however, empirical and calibration-based: it does not develop a surface kinetic or thermodynamic model linking the atomic-to-molecular sticking probability ratio to the electrochemical equilibrium or steady-state flux of a working oxygen pump, nor does it address plasma-generated atomic oxygen specifically. The present work is, to our knowledge, the first to place this differential sticking behavior on a quantitative kinetic footing, via the surface reaction model of eqs 8 and 9, and to use it to explain and predict plasma-enhanced oxygen pumping performance in an SOEC.

While the plasma also generates electronically excited molecular oxygen species, their contributions to the surface oxygen coverage balance are considered to be negligible under the present conditions. The singlet sigma state O_2_(b^1^Σ_g_^+^) is produced at significantly lower densities than atomic oxygen and undergoes rapid collisional de-excitation in the afterglow;^31^ at our operating pressure of 1 Torr, its production density is proportionally lower, making its contribution to surface oxygen coverage negligible. The singlet delta state O_2_(a^1^Δ_g_) is longer-lived but its reactivity advantage over ground-state O_2_, arising from its 0.98 eV electronic excitation energy lowering the dissociative adsorption barrier, is expected to diminish substantially at the SOEC operating temperatures of 692 °C (965 K), where the surface thermal energy is high. The quantitative agreement of the two-species surface reaction model with the measured OCV transients at two independent plasma power conditions further confirms that no additional reactive species contribute meaningfully to the surface oxygen coverage balance. Atomic oxygen is therefore considered to be the dominant plasma-generated species responsible for the enhanced ORR kinetics. The interpretation of the OCV shift as a change in cathode surface oxygen chemical potential is supported by several mutually consistent lines of evidence, and alternative origins can be excluded on physical grounds. Electrostatic surface charging would produce a response on the RC time scale of the LSM electrode: with an electronic conductivity of order 30–150 S/cm and a true interfacial double-layer capacitance of order 10^–6^ F/cm^2^ at the LSM/YSZ interface,^4^ the RC discharge time is at most of order 10^–7^ s, more than 7 orders of magnitude faster than the observed 40–80 s transient. Modification of the LSM/YSZ interfacial double-layer potential would operate on the ionic relaxation time scale of YSZ at our operating temperature (milliseconds to seconds), similarly inconsistent with the observed time scale, and would not produce quantitative Nernst scaling with the measured atomic oxygen flux. Direct plasma-SOEC electrical coupling is excluded by the impedance-to-ground exceeding 10 MΩ. The characteristic fitted time scale 1/|*k*_2_| is constant across both plasma power conditions (Tables S1 and S2), confirming that it reflects a surface property of LSM independent of plasma power. The plasma-on transient is systematically faster than the plasma-off transient (τ_on_ ≈ 13–17 s, τ_off_ ≈ 22–23 s) at both power conditions, consistent with the rate equation prediction that the plasma-on approach is accelerated by the additional atomic oxygen adsorption term Γ_O_ while plasma-off relaxation proceeds through the molecular oxygen pathway alone. This specific combination of power-independent time scale, plasma-on/off asymmetry, full reversibility, and quantitative Nernst scaling with independently measured atomic oxygen flux collectively confirm that the OCV shift originates from enhanced atomic oxygen adsorption at the LSM cathode surface.

To assess the effect of plasma on oxygen pumping performance, the external applied voltage *V*_SOEC_ is held constant while the plasma is cycled on and off. Figure 5 presents a typical time-sequence measurement of plasma-enhanced SOEC oxygen pumping for varying plasma power levels (*P*_plasma_) of 494, 366, and 250 W, as indicated by red, green, and blue, respectively. Without plasma, the SOEC current stabilizes at approximately 0.8 mA, corresponding to an oxygen flux Φ_O_2__ of 2.8 × 10^–3^ mln/min. Upon plasma ignition, the current increases monotonically with plasma power: approximately 1.0 mA at 250 W, 1.2 mA at 360 W, and 1.3 mA at 490 W. At maximum power, the oxygen flux Φ_O_2__ rises to 4.9 × 10^–3^ mln/min, a 75% increase under constant applied voltage. Upon plasma extinction, the oxygen flux returns reproducibly to its baseline value across all three cycles, confirming the reversibility of the plasma enhancement. Minor current fluctuations are attributed to SOEC temperature variations of within ±3.5 K arising from the temperature control feedback loop.

**Figure 5:**
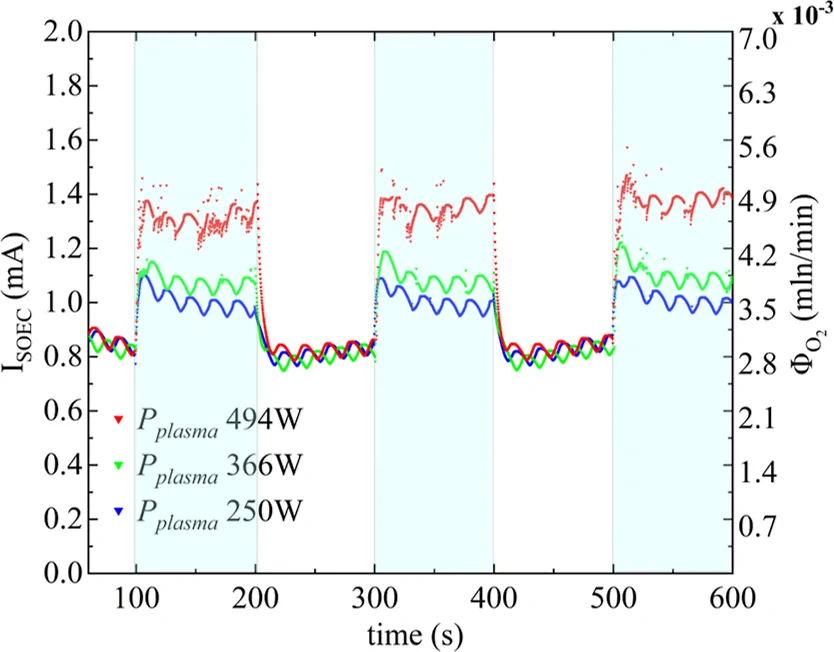
Oxygen pumping experiment with 1 Torr He–O_2_ plasma (*p*O_2_ 0.2 Torr, 10 sccm). The applied voltage *V*_SOEC_ is fixed to −0.25 V, *T*_SOEC_ 652 °C and the time regions with plasma-on are marked in green.

To enable quantitative comparison across operating conditions, we define a relative plasma enhancement metric κ

10 $$\kappa =\frac{\Delta \Phi_{O_{2}}}{\Phi_{O_{2}}^{noplasma}}=\frac{\Phi_{O_{2}}^{plasmaon}-\Phi_{O_{2}}^{noplasma}}{\Phi_{O_{2}}^{noplasma}}$$

The dependence of κ on SOEC temperature *T*_SOEC_, applied voltage *V*_SOEC_, and plasma power *P*_plasma_ is summarized in Figure 6.

**Figure 6:**
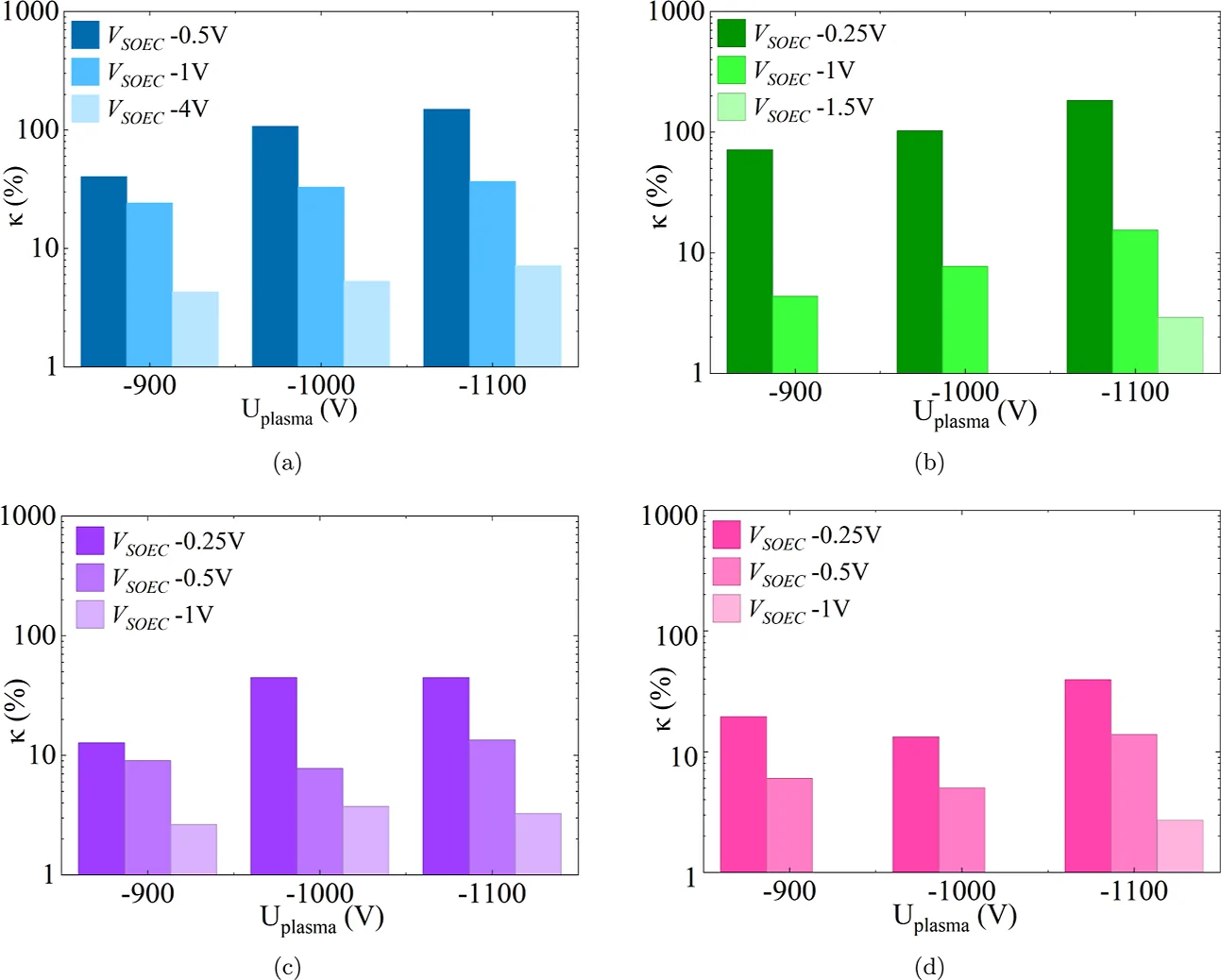
Plasma enhancement effect κ on the SOEC oxygen-pumping flux in 1 Torr He–O_2_ plasma at intermediate temperatures: (a) *T*_SOEC_ = 494 °C, *P*_plasma_ = 247/357/465 W; (b) *T*_SOEC_ = 585 °C, *P*_plasma_ = 247/355/470 W; (c) *T*_SOEC_ = 652 °C, *P*_plasma_ = 250/366/494 W; and (d) *T*_SOEC_ = 692 °C, *P*_plasma_ = 224/337/456 W. For each panel, *U*_plasma_ = −900/–1000/–1100 V, respectively.

The plasma enhancement is strongly temperature-dependent, with the largest κ values observed below 600 °C. At *T*_SOEC_ 585 °C, with an *V*_SOEC_ −0.25 V and *P*_plasma_ of 470 W, the oxygen flux increases remarkably by approximately 180%, from 1.85 × 10^–3^ mln/min to 5.24 × 10^–3^ mln/min. At *T*_SOEC_ 692 °C under otherwise comparable conditions (456 W, −0.25 V), the flux increases from 6.11 × 10^–3^ mln/min to 8.53 × 10^–3^ mln/min, a 39% increase. This temperature dependence is consistent with the plasma effect operating through surface kinetics: at lower temperatures, intrinsic ORR surface rates are slower, and plasma-supplied atomic oxygen has proportionally greater impact on the adsorption-limited step. However, the enhancement does not increase strictly monotonically with decreasing temperature, with the largest enhancement ratios observed around 585 °C rather than at the lowest temperature investigated (494 °C). This reflects two competing effects: below 585 °C, the thermally activated electrode exchange kinetics and YSZ ionic transport become sufficiently slow to limit the efficiency with which additional surface coverage is converted into electrochemical current, while the baseline coverage θ_0_ under purely molecular oxygen conditions increases toward saturation owing to the exothermic nature of oxygen adsorption,^14^ reducing the headroom for plasma-supplied atomic oxygen; above 585 °C, the baseline current grows rapidly with temperature, so the power-fixed atomic oxygen flux represents a diminishing fractional contribution. This nonmonotonic behavior is most pronounced at small applied voltages, where the current is most sensitive to changes in surface oxygen activity, and diminishes at higher voltages where ohmic resistance through the YSZ electrolyte dominates the cell response.

The enhancement also depends strongly on the operating regime. In the activation-loss-dominated region, plasma reduces *E*_OCV_ by increasing cathode surface oxygen activity, which increases the net electrochemical driving force *V*_SOEC_ – *E*_OCV_ and amplifies the activation overpotential available for driving the ORR. Since ohmic and concentration losses are negligible in this region, even a modest increase in surface oxygen coverage translates directly into improved reaction kinetics and oxygen flux. In the ohmic region, where the *I*–*V* response is approximately linear and cell performance is dominated by ionic resistance through the YSZ electrolyte (see Figure S1), plasma-induced changes in surface coverage have no meaningful effect on oxygen flux. Accordingly, κ falls below 10% at the highest applied bias for all temperatures tested, and at *T*_SOEC_ 585 and 692 °C the values are slightly negative (ranging from −0.0043% to −0.56%), indicating that plasma provides no net benefit when bulk resistance dominates. These near-zero and negative values are excluded from the logarithmic plot.

The enhancement scales monotonically with plasma power, consistent with the higher atomic oxygen production at elevated discharge power established in the OCV analysis. However, the efficiency of atomic oxygen utilization is low: the incremental oxygen flux through the SOEC (ΔΦ_O_2__) is of order 10^17^ to 10^19^ m^–2^·s^–1^, while the atomic oxygen flux delivered from the plasma to the cathode is of order 10^20^ to 10^21^ m^–2^·s^–1^. Only approximately 0.1% of plasma-delivered atomic oxygen contributes to electrochemical oxygen transport, indicating that bulk transport through the electrolyte, rather than surface adsorption, is the dominant bottleneck under the present conditions of thick electrolyte (1.5 mm) and intermediate operating temperature.

## Conclusions

In summary, the plasma-induced enhancement of oxygen pumping is most pronounced in the activation loss dominated region, particularly near OCV conditions, consistent with plasma-driven enhancement of surface oxygen adsorption kinetics rather than bulk transport. At *p*O_2_ 0.2 Torr, *T*_SOEC_ 585 °C and *P*_plasma_ 470 W, coupling the glow discharge to the SOEC cathode yields a 180% increase in oxygen pumping capacity relative to conventional SOEC operation. Performance in the ohmic loss region remains governed by SOEC configuration and operating conditions, including electrolyte thickness and applied voltage, confirming that plasma activation acts through surface rather than bulk pathways.

A surface reaction model fitted to OCV transients in He–O_2_ plasma separates the contributions of molecular and atomic oxygen fluxes to cathode surface coverage. The model yields a ratio of atomic-to-molecular oxygen sticking probabilities $\frac{\gamma_{O}}{\gamma_{O_{2}}}$ = 286 (±110) at *p*O_2_ 0.2 Torr, *T*_SOEC_ 692 °C. The flux ratio extracted from OCV measurements agrees quantitatively with direct oxygen radical measurements, validating the model and identifying plasma-generated atomic oxygen as the dominant species responsible for enhanced ORR kinetics. Together, these results demonstrate that nonequilibrium plasma selectively targets the dissociative adsorption step that rate-limits conventional SOEC operation, and that the degree of enhancement is predictable from measurable plasma flux parameters. This mechanistic understanding provides a basis for designing plasma-assisted solid oxide oxygen separation systems, where surface kinetics rather than bulk transport are the controlling factor.

## Experimental Methods

The SOEC is positioned in the afterglow region of four glow discharges, as shown in Figure 7. The plasma feed gas is a He–O_2_ mixture (80% He, 20% O_2_) supplied at 10 sccm via a mass flow controller (Bronkhorst) at a total pressure of 1 Torr, monitored by a vacuum transducer (MKS 974B). All four plasma cathodes are biased to the same negative voltage by a high-voltage DC supply (FUG MCA 750–1500). The discharge volume is occupied by the negative glow region and no positive column present.^32^ The inverse brush cathodes have a diameter of 54 mm and contain 95 evenly spaced holes (diameter 3 mm, depth 25 mm, center-to-center spacing 5 mm) drilled into the cathode face; the cathodes are water-cooled. The cathode-to-anode gap is 60 mm in each case. Four Pyrex tubes (outer diameter 65 mm, wall thickness 3 mm) are installed between the cathodes and the central anode ring to provide electrical isolation and confine the gas flow. Plasma power is computed as *P*_plasma_ = *U*_plasma_ × *I*_plasma_, where voltage and current are read directly from the power supply during steady-state DC discharge. Electron density, electron temperature, and atomic oxygen density are measured in the afterglow region a few millimeter from the SOEC surface. SOEC current–voltage characteristics and OCV transients are recorded with a potentiostat (IviumStat.XRe) powered through an isolation transformer. The SOEC has no direct electrical connection to the plasma ground anode, and its impedance to ground, measured with a multimeter, exceeds 10 MΩ, confirming that the SOEC floats electrically relative to the plasma circuit.

**Figure 7:**
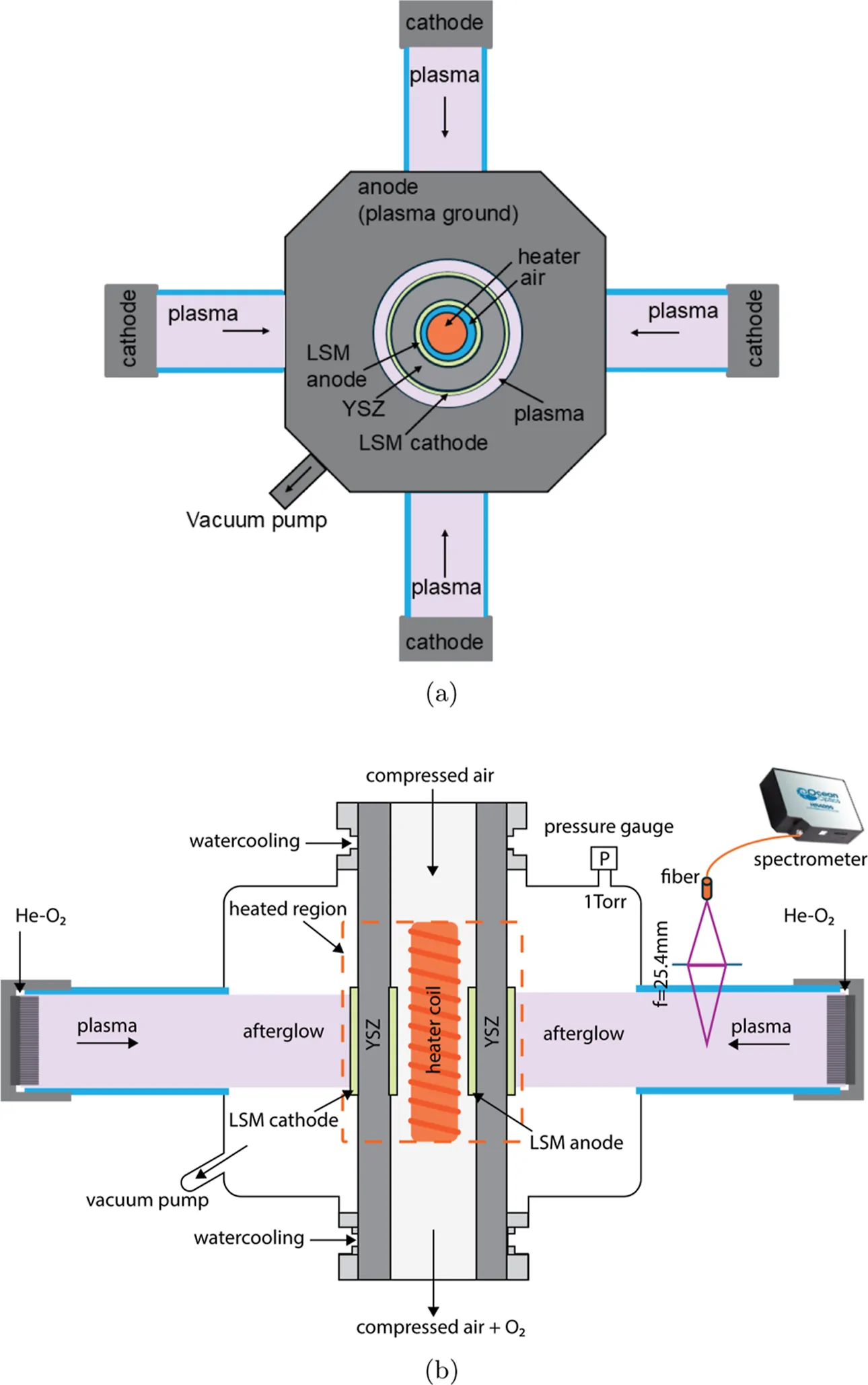
Schematic view of the plasma-SOEC setup. (a) Top view showing the radial arrangement of the four glow discharge cathodes around the central reactor body (anode, plasma ground), the direction of plasma flow toward the SOEC, and the vacuum pump outlet. The central radial layer structure of the SOEC tube proceeds outward from a resistive heater at the core, through an air gap and the LSM anode on the inner tube wall, across the YSZ electrolyte, to the LSM cathode on the outer wall, which is in turn surrounded by the plasma afterglow. (b) Axial cross-sectional view showing the heated region, YSZ tube, LSM electrodes and plasma afterglow regions.

The LSM|YSZ|LSM symmetric cell was fabricated from a yttria-stabilized zirconia (YSZ) electrolyte tube (Gimex, The Netherlands; outer diameter 30 mm, wall thickness 1.5 mm) with concentric inner and outer (La_0.8_Sr_0.2_)_0.95_MnO_3−δ_ (LSM) electrodes. LSM electrode ink (Fuelcellmaterials, USA) was brush-painted onto the outer and inner surfaces of the YSZ tube, each electrode spanning 50 mm. The assembly was sintered at 1100 °C for 6 h to burn out the organic binder and bond the LSM to the YSZ surface. A silver ink layer was subsequently applied over each LSM electrode and cured at 600 °C for 6 h to improve electronic contact and current distribution.^33^ Silver meshes were pressed against the silver ink layers and secured with silver wire to serve as current collectors. The role of the silver layer under the present experimental conditions warrants clarification. Bulk Ag_2_O is thermally unstable above approximately 380 °C and decomposes to metallic silver and O_2_; at the SOEC operating temperatures of 585–692 °C used here, the silver layer therefore remains metallic rather than forming a stable bulk oxide phase. The silver surface acts as a current-collecting interface that remains active for oxygen adsorption and surface exchange.^33,34^ Under plasma exposure, atomic oxygen delivered to the Ag/LSM cathode surface provides an additional adsorbed oxygen flux, increasing surface coverage θ_O_ and cathode-side oxygen activity. This surface mechanism accounts for the observed OCV shift and requires no bulk silver oxide formation. The silver mesh used as current collector has a 40 mesh × 0.11 mm wire diameter specification, yielding an open area fraction of approximately 68% and a wire contact area fraction of approximately 32% of the total geometric electrode area. The total geometric area of the outer (cathode) LSM electrode is approximately 47 cm^2^, giving an exposed LSM/Ag ink area of approximately 32 cm^2^ and an Ag wire contact area of approximately 15 cm^2^. Since the silver ink layer is applied over the full electrode surface prior to mesh placement, the functional current-collecting interface encompasses the full electrode area. The LSM electrode thickness was not directly measured; the electrode was applied by brush painting in 2 coats using commercial LSM ink (Fuelcellmaterials, USA) sintered at 1100 °C for 6 h. The total sintered electrode thickness in the present work is estimated to be in the range of 80–150 μm. Cross-sectional SEM characterization in future work would allow this to be established more precisely.

The SOEC was sealed in the plasma reactor via a flange system with water-cooled O-ring seals. The cell temperature was controlled by a resistive heating coil positioned inside the YSZ tube. A temperature calibration was performed in a tubular furnace to establish the Arrhenius relationship between YSZ bulk ionic conductivity and temperature; this calibration was used to determine the SOEC temperature in the plasma reactor from conductivity measurements. The in situ conductivity measurement integrates over the full 1.5 mm YSZ wall thickness and therefore yields the volume-average bulk electrolyte temperature rather than the electrode surface temperature directly. To assess whether plasma-induced heating introduces a meaningful radial temperature gradient between the plasma-facing outer LSM cathode surface and the inner anode surface, two independent estimates of the heat deposited by atomic oxygen recombination were performed. A conservative plasma power budget gives an upper bound of *Q* ≤ 14 W, while a realistic bottom-up calculation using the experimentally measured atomic oxygen flux (Supporting Information, Figure S6) and literature recombination probabilities for Ag and LSM yields *Q* = 0.007–0.04 W. Applying the cylindrical heat conduction formula with the YSZ geometry (*r*_out_ = 15 mm, *r*_in_ = 13.5 mm, *k* = 2.0 W m^–1^ K^–1^, *L* = 50 mm) gives a radial temperature gradient of Δ*T* ≤ 0.007 K under realistic conditions and Δ*T* ≤ 2.4 K under the conservative upper bound. Both estimates confirm that plasma-induced thermal gradients are negligible relative to the ±3.5 K temperature stability of the resistive heating system and to the 40–91 K temperature steps between the operating conditions compared in Figure 6. The plasma enhancement is therefore attributed exclusively to plasma-chemical surface effects rather than bulk thermal perturbation. Compressed air was supplied to the inner electrode compartment (anode side) at atmospheric pressure, while the outer electrode (cathode, plasma-facing side) was exposed to the He–O_2_ plasma at a total pressure of 1 Torr.

## Supplementary Material



## Notes

Additional experimental details of the SOEC performance, model derivations of open cell voltage in equilibrium and under plasma exposure, fitting functions for OCV transient response, and plasma radical and ion fluxes measurements (PDF)
