# Supplementary material for: Plasma-Enhancement of Oxygen Pumping: The Role of Atomic Oxygen
Source: J Phys Chem C Nanomater Interfaces. 2026 Aug 24;130(42):14465–73. doi: 10.1021/acs.jpcc.6c02560 (PMC13700085; doi:10.1021/acs.jpcc.6c02560)
Supplement: Supplementary file 1 [file jp6c02560_si_001.pdf]

Supporting Information for

Plasma-Enhancement of Oxygen Pumping: The Role of Atomic Oxygen

Xingyu Chen*[1,2], Aleksandr Pikalev[2], Susanta Bera[2], Mihalis N. Tsampas[2], Guan-Jun Zhang[1], and M.C.M. van de Sanden*[2,3]

[1]School of Electrical Engineering, Xi'an Jiaotong University, Xi'an, Shaanxi 710049, China
[2]Dutch Institute for Fundamental Energy Research (DIFFER), De Zaale 20, 5612 AJ Eindhoven, The Netherlands
[3]Eindhoven Institute for Renewable Energy Systems (EIRES), Eindhoven University of Technology, 5612 AZ Eindhoven, The Netherlands

*Email: x.chen@differ.nl, M.C.M.vandeSanden@differ.nl

S1. Experimental Details

Representative I-V characteristics of the SOEC at varying cathode-side oxygen partial pressures are shown in Figure S1(a), with the applied voltage referenced to OCV to give the overpotential η in Figure S1(b). At low overpotentials ($|\eta| < 0.25$ V), the approximately linear dependence in the ln I-η indicates Tafel-like behavior, consistent with activation-controlled electrode kinetics. In the intermediate range (approximately -0.25 to -1.0 V), the corresponding I-V curves (inset, Figure S1(b)) become linear on a linear scale, indicating that the cell response is dominated by ohmic resistance, primarily

associated with ionic transport through the YSZ electrolyte. At higher overpotentials ($|\eta|$ >1.0 V), the current continues to increase approximately linearly with overpotential, consistent with ohmic-dominated transport through the YSZ electrolyte. A marginal reduction in the rate of current increase is observed only at the highest overpotentials for the lowest pO_2 condition tested (0.1 Torr), suggesting the onset of concentration polarization under the most oxygen-limited conditions. The plasma enhancement experiments are conducted in the activation and ohmic regimes, where surface kinetics and electrolyte resistance are the respective limiting factors.

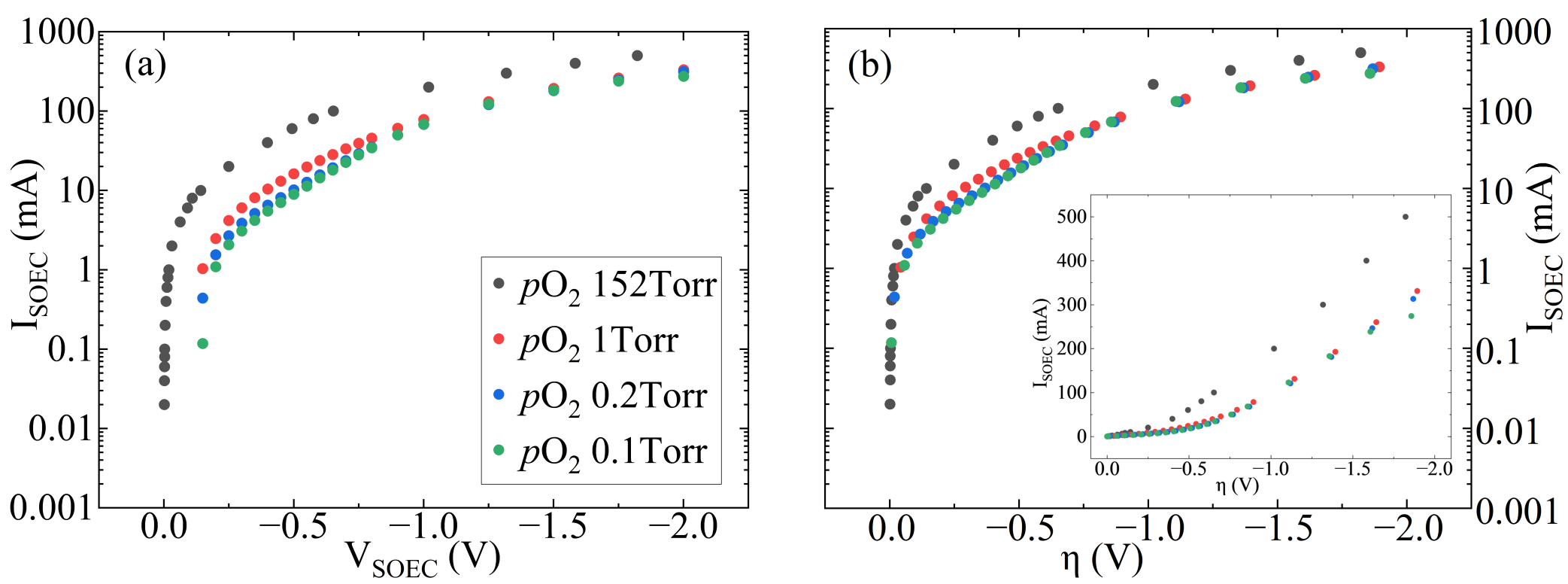

Figure S1: SOEC polarization curves (without plasma exposure) with different cathode-side pO_2 at T_{SOEC} 692°C: (a) $I_{SOEC} - V_{SOEC}$ curve and (b) I_{SOEC}–η curve. The measurement of pO_2 152 Torr corresponds to the oxygen partial pressure and was conducted in air, and the measurements of pO_2 0.1-1 Torr were conducted in He-O_2.

The exchange current density is conventionally extracted from the pO_2 dependence of the current near OCV, either from I-V measurements at low overpotential or from electrochemical impedance spectroscopy.[1] Here, the SOEC current at a fixed applied voltage close to OCV is used as a proxy for the exchange current. The dependence of I_{SOEC} on pO_2 at three applied voltages (−0.5, −1.0, −1.5 V) is shown in Figure S2 on a double-logarithmic scale. At -0.5 V, which most closely approximates OCV conditions, the power-law exponent is 0.263 (± 0.005). This value is consistent with low fractional oxygen surface coverage on the LSM electrode under the present experimental conditions, in agreement with the mechanistic interpretation reported for LSM cathodes at comparable temperatures and oxygen partial pressures.[1,2]

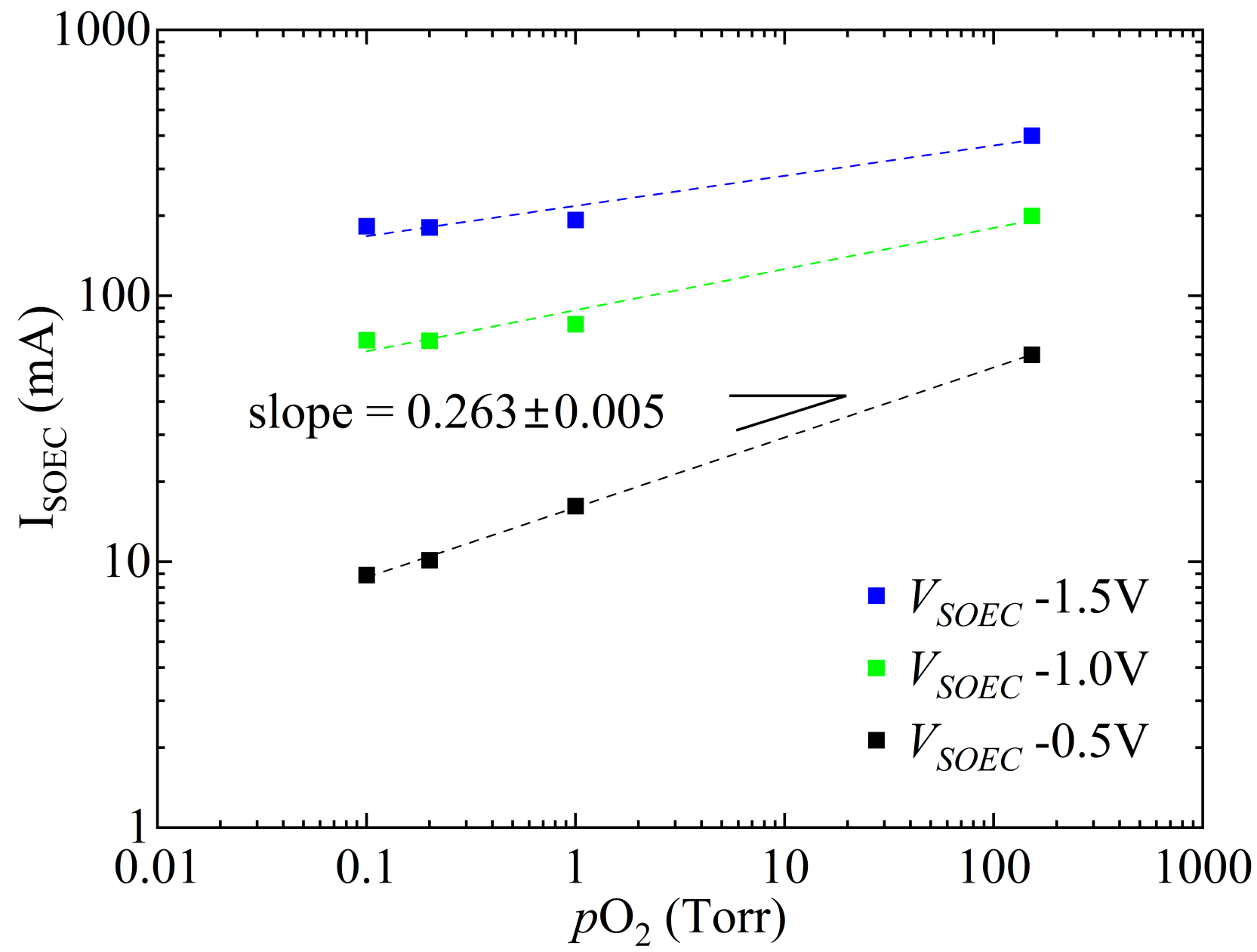

Figure S2: Comparison of $\log(I_{\mathrm{SOEC}})$ vs $\log(pO_2)$ under different conditions.

S2. Open cell voltage in equilibrium and under plasma exposure

The SOEC geometry and coordinate convention used in the derivation are defined in Figure S3. The outer LSM electrode, exposed to He-O_2 plasma at 1 Torr, is designated the cathode at x=0; the inner LSM electrode, exposed to ambient air at pO_2 =152 Torr, is designated the anode at x=L. As described in the Experimental section of the main manuscript, the OCV was recorded under zero-current conditions using a high-impedance potentiostat, ensuring negligible current draw, and the SOEC was electrically floating relative to the plasma circuit and vacuum vessel throughout all measurements, as confirmed by the impedance-to-ground exceeding 10 MΩ reported in the experimental section. This information is briefly restated here so that the derivation in this section can be followed without requiring cross-reference to the main text. The voltage U measured by the potentiostat is the difference between the electrical potentials φ at the cathode and anode electrode:

$$U = \varphi_0 - \varphi_L \tag{S1}$$

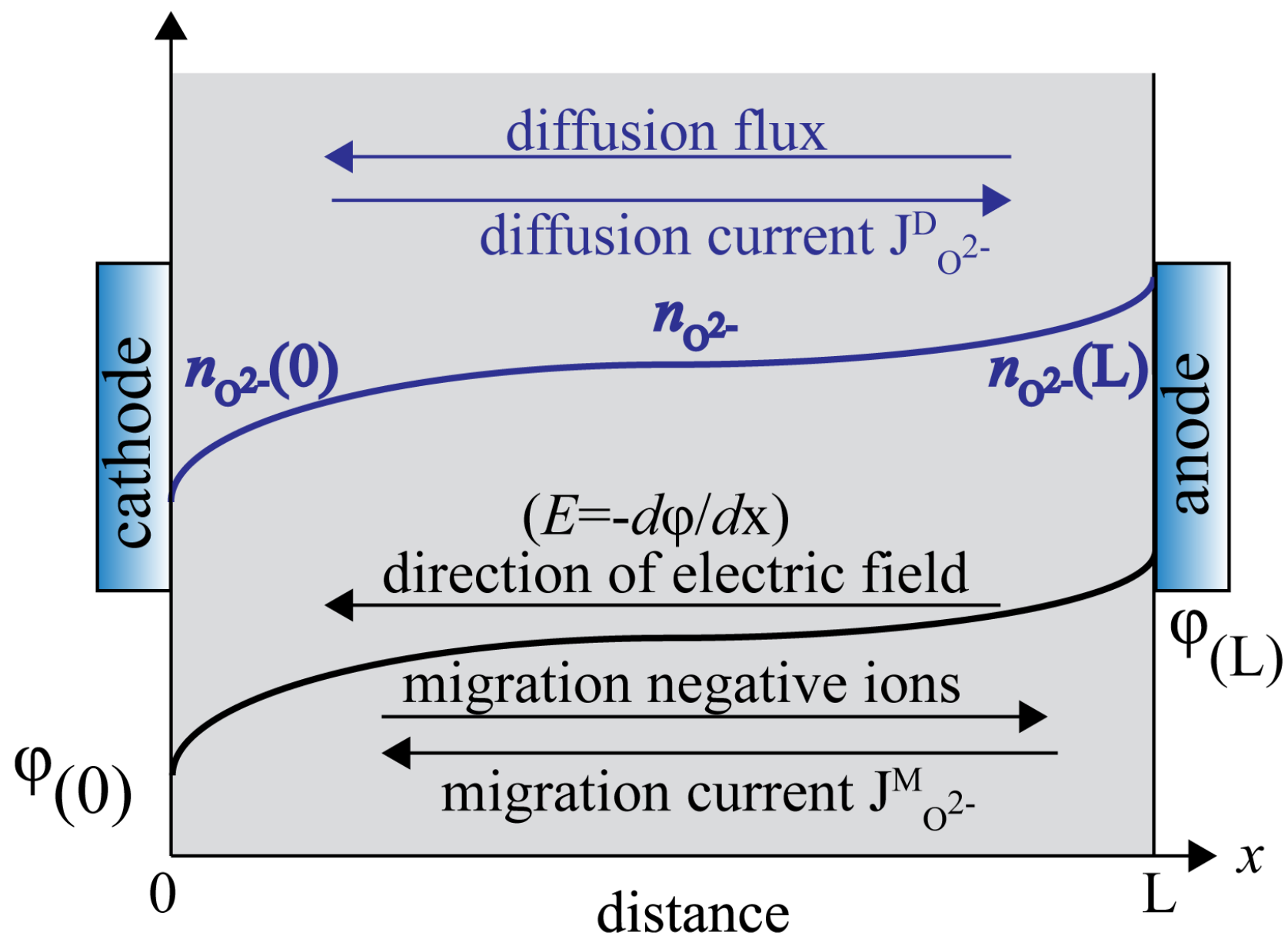

Figure S3: Schematic illustration of the qualitative trends in oxygen ion number density $n_{O^{2-}}$ and electrical potential φ across the YSZ electrolyte under open-circuit conditions. The profiles are representative of the expected physical behavior and are intended to define the coordinate convention used in the derivation of S2; they are not derived from direct measurement or numerical calculation.

Note that U denotes the general electrical potential difference across the SOEC electrodes. Under open-circuit conditions ($I = 0$), U reduces to E_{OCV} as derived in the remainder of this section, while under applied voltage conditions U corresponds to V_{SOEC} as used in Figure S1. Under open circuit conditions ($I = 0$), $U = E_{OCV}$, determined by the difference in electrochemical potential $\tilde{\mu}$ between the cathode and anode. The electrochemical potential of species i is expressed as the sum of its chemical potential μ and the molar electrostatic potential energy:

$$\tilde{\mu}_i = \mu_i + z_i F\Phi = \mu_i^0 + RT\ln a_i + z_i F\Phi \tag{S2}$$

where μ_i^0 is the standard chemical potential of the species i (J mol^{-1}), a_i is its dimensionless activity, z_i is the charge number of species i (dimensionless), R (gas constant, 8.314 J mol^{-1} K^{-1}), T (absolute temperature, K), F is the Faraday constant (96485 C mol^{-1}), and Φ is the local electrostatic potential (V). For neutral species, such as O_2, there is no charge (z_i=0), so the electrochemical potential is equal to the chemical potential:

$$\tilde{\mu}_{O_2} = \mu_{O_2} = \mu_{O_2}^0 + RT\ln(a_{O_2}) \tag{S3}$$

For the O^{2-}, the main charge carrier in YSZ, the electrochemical potential is given by:

$$\tilde{\mu}_{O^{2-}} = \mu_{O^{2-}} - 2F\Phi \tag{S4}$$

The electrochemical potential for the electrons is defined as:

$$\tilde{\mu}_{e^-} = \mu_{e^-} - F\Phi \tag{S5}$$

The electrochemical equilibrium conditions at the cathode and anode are respectively:

$$\frac{1}{2}\mathrm{O_2}(0) + 2\mathrm{e}^-(0) \rightleftharpoons \mathrm{O}^{2-}(0) \; i.e. \; \frac{1}{2}\tilde{\mu}_{O_2}(0) + 2\tilde{\mu}_{e^-}(0) = \tilde{\mu}_{O^{2-}}(0) \tag{S6}$$

$$\frac{1}{2}\mathrm{O_2}(L) + 2\mathrm{e}^-(L) \rightleftharpoons \mathrm{O}^{2-}(L) \; i.e. \; \frac{1}{2}\tilde{\mu}_{O_2}(L) + 2\tilde{\mu}_{e^-}(L) = \tilde{\mu}_{O^{2-}}(L) \tag{S7}$$

where Eq. (S6) describes the cathode reaction at low pO_2 (plasma reactor, 0.2 Torr) and Eq. (S7) describes the anode reaction in ambient air at fixed pO_2 152 Torr. In both equations, $O_2(0)$ and $O_2(L)$ refer to the gas phase oxygen, $O^{2-}(0)$ and $O^{2-}(L)$ refer to oxygen ions at the electrolyte-electrode interfaces,

and $e^-(0)$ and $e^-(L)$ refer to electrons in the respective electrode layers. Considering O^{2-} as the only ion charge carrier in the YSZ electrolyte, the condition of uniform electrochemical potential across the electrolyte requires:

$$\tilde{\mu}_{O^{2-}}(0) = \tilde{\mu}_{O^{2-}}(L) \tag{S8}$$

Combining Eqs.(S6)–(S8), the OCV is:

$$E_{OCV} = \frac{RT}{4F} \ln \frac{a_{O_2}(0)}{a_{O_2}(L)} = \frac{RT}{2F} \ln \frac{a_{O^{2-}}(0)}{a_{O^{2-}}(L)} \tag{S9}$$

where the factor of 2 difference in the prefactors arises because, under adsorption equilibrium, $a_{O_2} \propto a_{O^{2-}}^2$.

Under chemical equilibrium and ideal gas conditions, $a_{O_2} \approx pO_2/p^O$, and Eq. (S9) reduces to the classical Nernst equation:

$$E_{OCV} = \frac{RT}{4F} \ln \frac{pO_2(0)}{pO_2(L)} \tag{S10}$$

where $pO_2(0)$ and $pO_2(L)$ are the oxygen partial pressures at the cathode and anode, respectively. Since $pO_2(0) \ll pO_2(L)$, E_{OCV} takes a negative value under the present experimental conditions.

However, Equation (S10) is no longer valid when the cathode is exposed to non-equilibrium plasma fluxes, as the assumption of gas-phase oxygen activity breaks down. A generalized expression for E_{OCV} in terms of electrode surface oxygen coverage needs to be derived.

Whereas the first part of Eq. (S9), expressed in terms of gas-phase oxygen activities, is valid only under thermodynamic equilibrium, the second part, expressed in terms of O^{2-} activities at the electrolyte-electrode interfaces, remains valid under generalized non-equilibrium conditions provided local interfacial equilibrium is maintained.[3] Under this condition, the O^{2-} activity $a_{O^{2-}}$ is identified with the O^{2-} number density $n_{O^{2-}}$ at each electrolyte-electrode interface. Since the O^{2-} density at the interface is proportional to the surface adsorbed oxygen density $n_{O_{ad}}$ at the electrode, the OCV under generalized conditions, including non-equilibrium plasma exposure, is expressed in terms of $n_{O_{ad}}$ at each electrode:

$$E_{OCV} = \frac{RT}{2F} \ln \frac{n_{O^{2-}}(0)}{n_{O^{2-}}(L)} = \frac{RT}{2F} \ln \frac{n_{O_{ad}}(0)}{n_{O_{ad}}(L)} \tag{S11}$$

The anode-side surface coverage $n_{O_{ad}}(L)$ is governed by the adsorption-desorption equilibrium:[3]

$$O_2(g) + 2S \underset{K_d}{\overset{K_a}{\rightleftharpoons}} 2O_{ad} : S \tag{S12}$$

where K_a and K_d are the adsorption and desorption rate constants, both functions of electrode temperature. Since each O_2 molecule requires two surface sites, the adsorption and desorption fluxes are:

$$\Gamma_{ads} = K_a n_{O_2} n_0^{'2} (1-\theta)^2 \tag{S13}$$

and the molecular flux desorbed equals

$$\Gamma_{des} = K_d n_0^{'2} \theta^2 \tag{S14}$$

where n_{O_2} is the gas-phase O_2 density near the surface, $n_0^{'}$ is the areal density of adsorption sites, and θ is the fractional surface coverage. Setting $\Gamma_{ads} = \Gamma_{des}$ gives the equilibrium surface coverage: :

$$\theta = \frac{(n_{O_2} \cdot K_a/K_d)^{1/2}}{1 + (n_{O_2} \cdot K_a/K_d)^{1/2}} \tag{S15}$$

The O_2 flux incident on the surface is given by $\Gamma_{O_2} = \frac{1}{4}\bar{v}_{O_2} n_{O_2}$, where $\bar{v}_{O_2}$ is the mean molecular speed. The adsorbed flux is equivalently expressed as:

$$\Gamma_{ads} = \gamma \Gamma_{O_2} = \frac{1}{4}\gamma \bar{v}_{O_2} n_{O_2} \tag{S16}$$

where γ is the dissociative adsorption sticking coefficient, which is a function of the surface coverage θ and electrode temperature. Combining Eqs (S13) and (S16) yields the relation $K_a = \frac{\gamma_{O_2}\bar{v}_{O_2}}{4n_O^{'2}(1-\theta)^2}$, linking the kinetic rate constant K_a to the sticking coefficient γ_{O_2}, which can in principle be determined independently, for example, via the King–Wells molecular beam method for measuring initial sticking probabilities on well-defined surfaces,[4] or via the $^{18}O/^{16}O$ isotope exchange depth profile (IEDP) technique combined with SIMS for oxide electrodes at elevated temperatures,[5] but is not directly measured in the present work. The analysis here instead extracts the ratio γ_O/γ_{O_2} from OCV transient fitting combined with direct flux measurements, which is the physically relevant quantity for quantifying the plasma enhancement.

In the absence of plasma, the same adsorption-desorption equations apply at the cathode, with $n_{O_{ad}}(0)$ replacing $n_{O_{ad}}(L)$ to reflect the lower cathode-side oxygen density.

S3. Fitting functions for OCV transient response

At steady state ($\frac{dn_{\mathrm{O_{ad}}}}{dt} = 0$) at the LSM cathode surface, Eq.(8) becomes:

$$\begin{aligned}\frac{\mathrm{d}n_{\mathrm{O_{ad}}}}{\mathrm{d}t} &= A - B \cdot n_{\mathrm{O_{ad}}}^2 \\ &= A\left(1 - \sqrt{\frac{B}{A}} \cdot n_{\mathrm{O_{ad}}}\right)\left(1 + \sqrt{\frac{B}{A}} \cdot n_{\mathrm{O_{ad}}}\right) = 0 \end{aligned} \tag{S17}$$

where $A = \Gamma_{\mathrm{O_2}}\gamma_{\mathrm{O_2}}$ is the adsorption term and $B = K_{des}$ is the desorption term. Under plasma-on conditions, Eq. (9) takes the analogous form with $A_p = \Gamma_{\mathrm{O_2}}\gamma_{\mathrm{O_2}} + \Gamma_{\mathrm{O}}\gamma_{\mathrm{O}}$ and $B_p = K_{des}$:

$$\begin{aligned}\frac{\mathrm{d}n_{\mathrm{O_{ad}}}}{\mathrm{d}t} &= A_p - B_p \cdot n_{\mathrm{O_{ad}}}^2 \\ &= A_p\left(1 - \sqrt{\frac{B_p}{A_p}} \cdot n_{\mathrm{O_{ad}}}\right)\left(1 + \sqrt{\frac{B_p}{A_p}} \cdot n_{\mathrm{O_{ad}}}\right) = 0 \end{aligned} \tag{S18}$$

Eq. (S17) is separated by partial fractions:

$$\left(\frac{1}{\left(1 - \sqrt{\frac{B}{A}} \cdot n_{\mathrm{O_{ad}}}\right)} - \frac{1}{\left(1 + \sqrt{\frac{B}{A}} \cdot n_{\mathrm{O_{ad}}}\right)}\right) \cdot \mathrm{d}n_{\mathrm{O_{ad}}} = 2A\mathrm{d}t \tag{S19}$$

Integrating both sides and applying the initial condition $n_{\mathrm{O_{ad}}}(t=0)$ to determine the integration constant C:

$$\sqrt{\frac{A}{B}}\left(-\ln\left(1-\sqrt{\frac{B}{A}}\cdot n_{\mathrm{O_{ad}}}(t)\right)+\ln\left(1+\sqrt{\frac{B}{A}}\cdot n_{\mathrm{O_{ad}}}(t)\right)\right)=2A\cdot t+C \tag{S20a}$$

$$\ln\frac{1+\sqrt{\frac{B}{A}}\cdot n_{\mathrm{O_{ad}}}(t)}{1-\sqrt{\frac{B}{A}}\cdot n_{\mathrm{O_{ad}}}(t)}=2\sqrt{AB}\cdot t+C \tag{S20b}$$

$$\frac{1+\sqrt{\frac{B}{A}}\cdot n_{\mathrm{O_{ad}}}(t)}{1-\sqrt{\frac{B}{A}}\cdot n_{\mathrm{O_{ad}}}(t)}=\mathrm{e}^{2\sqrt{AB}\cdot t+C} \tag{S20c}$$

Solving for $n_{\mathrm{O_{ad}}}(t)$:

$$n_{\mathrm{O_{ad}}}(t)=\sqrt{\frac{A}{B}}\cdot\frac{\mathrm{e}^{2\sqrt{AB}\cdot t+C}-1}{\mathrm{e}^{2\sqrt{AB}\cdot t+C}+1}=\sqrt{\frac{A}{B}}\cdot\frac{1-\mathrm{e}^{-C}\cdot\mathrm{e}^{-2\sqrt{AB}\cdot t}}{1+\mathrm{e}^{-C}\cdot\mathrm{e}^{-2\sqrt{AB}\cdot t}} \tag{S21}$$

An analogous derivation for the plasma-on case yields:

$$n_{\mathrm{O_{ad}}p}(t)=\sqrt{\frac{A_p}{B_p}}\cdot\frac{\mathrm{e}^{2\sqrt{A_pB_p}\cdot t+C_p}-1}{\mathrm{e}^{2\sqrt{A_pB_p}\cdot t+C_p}+1}=\sqrt{\frac{A_p}{B_p}}\cdot\frac{1-\mathrm{e}^{-C_p}\cdot\mathrm{e}^{-2\sqrt{A_pB_p}\cdot t}}{1+\mathrm{e}^{-C_p}\cdot\mathrm{e}^{-2\sqrt{A_pB_p}\cdot t}} \tag{S22}$$

Since the OCV is sensitive to the ratio of surface oxygen densities rather than their absolute values, the OCV data are normalized to their value at t=0. Using Eq. S11:

$$\frac{n_{\mathrm{O_{ad}}}(t)}{n_{\mathrm{O_{ad}}}(t=0)}=\exp\left(\frac{2F}{RT}\left(E_{\mathrm{OCV}}(t)-E_{\mathrm{OCV}}(t=0)\right)\right) \tag{S23a}$$

$$=\frac{1+\mathrm{e}^{-C}}{1-\mathrm{e}^{-C}}\cdot\frac{1-\mathrm{e}^{-C}\cdot\mathrm{e}^{-2\sqrt{AB}\cdot t}}{1+\mathrm{e}^{-C}\cdot\mathrm{e}^{-2\sqrt{AB}\cdot t}} \tag{S23b}$$

Eq. (S23b) is fitted to the normalized OCV data of Eq.(S23a), with the fitting function cast as:

$$\frac{n_{\mathrm{O_{ad}}}(t)}{n_{\mathrm{O_{ad}}}(t=0)}=\frac{\theta_{\mathrm{O}}(t)}{\theta_{\mathrm{O}}(t=0)}=k_1\cdot\frac{1-k_3\cdot\exp(k_2\cdot t)}{1+k_3\cdot\exp(k_2\cdot t)} \tag{S24}$$

where the fitting parameters are related to the physical quantities by:

$$k_1 = \frac{1+\mathrm{e}^{-C}}{1-\mathrm{e}^{-C}} = \sqrt{\frac{A}{B}} \cdot \frac{1}{n_{\mathrm{O_{ad}}}(t=0)} \tag{S25a}$$

$$k_2 = -2\sqrt{AB} = -2\sqrt{\Gamma_{\mathrm{O_2}}\gamma_{\mathrm{O_2}} \cdot K_{des}} \tag{S25b}$$

$$k_3 = \mathrm{e}^{-C} = \frac{n_{\mathrm{O_{ad}}}(t=\infty) + n_{\mathrm{O_{ad}}}(t=0)}{n_{\mathrm{O_{ad}}}(t=\infty) - n_{\mathrm{O_{ad}}}(t=0)} = \frac{\theta_{\mathrm{O}}(t=\infty) + \theta_{\mathrm{O}}(t=0)}{\theta_{\mathrm{O}}(t=\infty) - \theta_{\mathrm{O}}(t=0)} \tag{S25c}$$

Eqs. (S25a)-(S25c) apply to both plasma-on and plasma-off conditions. Note that the three unknowns $\gamma_{\mathrm{O_2}}$, γ_{O} and K_{des} cannot be independently determined from the fitting parameters alone; only their ratios are accessible, consistent with $k_1 = \frac{1+k_3}{1-k_3}$.By definition, $k_1 > 0$ and k_2 <0 (units: s^{-1}). The sign of k_3 is determined by the direction of surface coverage change: k_3 >0 when plasma ignition increases coverage ($\theta_{\mathrm{O}}(t=\infty) > \theta_{\mathrm{O}}(t=0)$), and k_3 <0 when plasma extinction decreases coverage ($\theta_{\mathrm{O}}(t=\infty) < \theta_{\mathrm{O}}(t=0)$). The surface reaction model is validated at different plasma power condition

Table S1: Fitting parameters for surface coverage response to He-O_2 plasma in Figure 4, U_{plasma} −1100 V, P_{plasma} 471 W

Parameter	Plasma-on	Plasma-off
k_1	1.9227 (±1.07×10^{-3})	0.5275 (±7.58×10^{-4})
k_2	-0.0747 (±3.7×10^{-4})	-0.0442 (±3.1×10^{-4})
k_3	0.3157 (±2.5×10^{-4})	-0.3094 (±6.5×10^{-4})

(U_{plasma} -1000 V, P_{plasma} 341 W). The model reproduces the measured OCV transients under both plasma-on and plasma-off conditions (Figure S4(a,b), fitting parameters in Table S2). Fitting yields $(\Gamma_{\mathrm{O_2}}\gamma_{\mathrm{O_2}} + \Gamma_{\mathrm{O}}\gamma_{\mathrm{O}}) \cdot K_{des}$= (8.732±0.013)×10^{-4} (s^{-2}) under plasma-on conditions and $\Gamma_{\mathrm{O_2}}\gamma_{\mathrm{O_2}} \cdot K_{des}$= (5.108±0.009)×10^{-4} (s^{-2}) under plasma-off conditions. The plasma-off term is consistent with the value of (4.882±0.007)×10^{-4} (s^{-2}) obtained at 471 W, with a difference of 4.6% between the two conditions. This agreement confirms that K_{des} depends primarily on SOEC temperature and is insensitive to plasma power over the range 341–471 W tested here.

The atomic-to-molecular oxygen flux ratio is calculated for U_{plasma} −1000 V (P_{plasma} 341 W). At 341 W, the averaged ion/electron flux is also three orders of magnitude lower than Γ_{O} and five orders of magnitude lower than

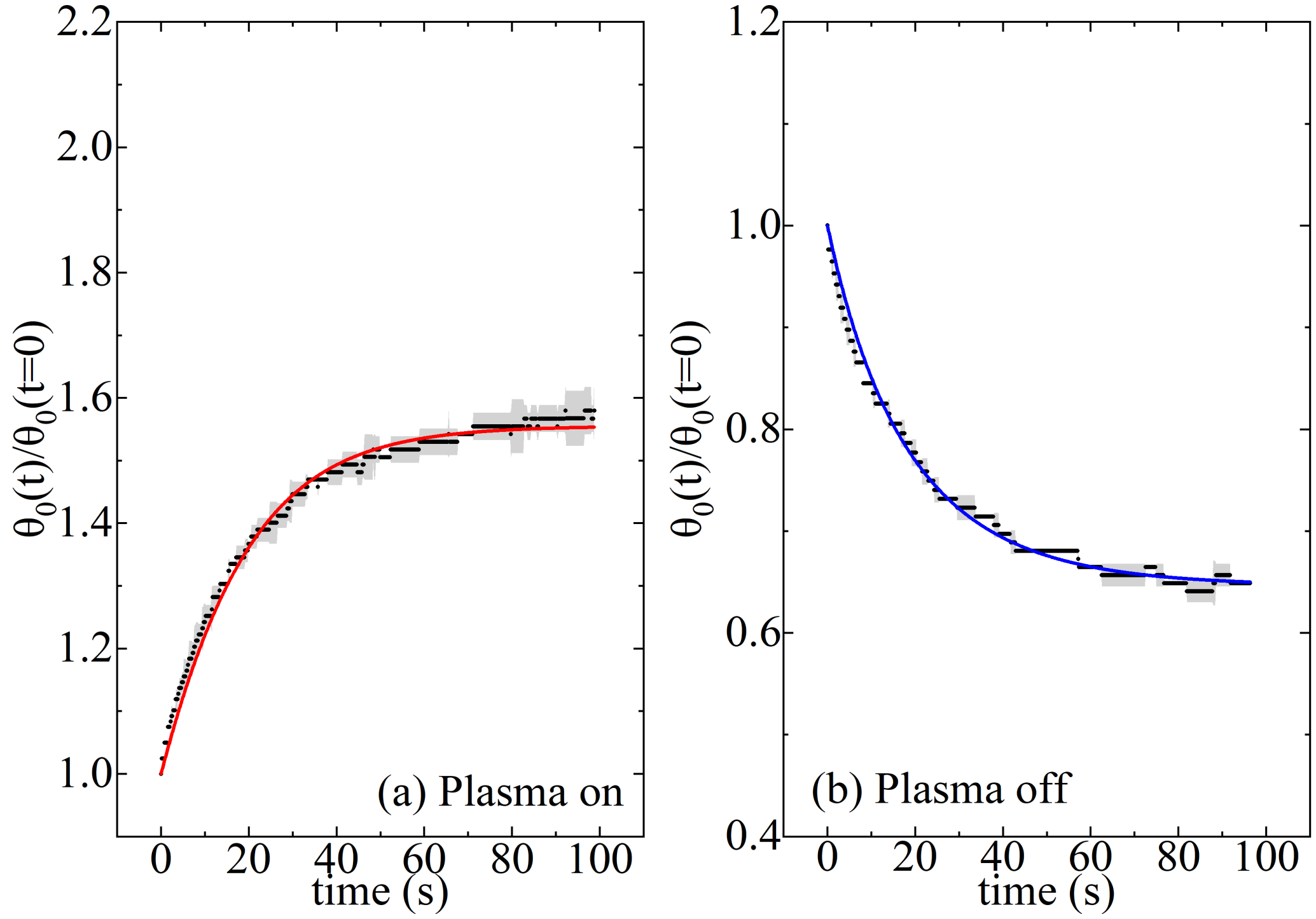

Figure S4: LSM cathode surface coverage response to 1 Torr He-O_2 plasma. Red and blue lines are the fitting curves for (a) plasma on and (b) plasma off. U_{plasma} -1000 V, P_{plasma} 341 W, pO_2 0.2 Torr, inlet flow 10 sccm, T_{SOEC} 692 °C. OCV data are averaged from 3 measurements, with standard deviation indicated by shading.

Table S2: Fitting parameters for surface coverage response to He-O_2 plasma in Figure S4, U_{plasma} -1000 V, P_{plasma} 341 W

Parameter	Plasma-on	Plasma-off
k_1	1.555($\pm 1.08\times10^{-3}$)	0.6464($\pm 7.7\times10^{-4}$)
k_2	-0.0591 ($\pm 4.3\times10^{-4}$)	-0.0452 ($\pm 3.9\times10^{-4}$)
k_3	0.2173 ($\pm 3.3\times10^{-4}$)	-0.2148 ($\pm 5.7\times10^{-4}$)

Γ_{O_2} under this plasma power conditions (Supporting Information, Fig.S6). Since K_{des} cancels in the ratio, the atomic-to-molecular oxygen flux ratio at U_{plasma} -1000 V (P_{plasma} 341 W) is calculated directly from the fitted products obtained under plasma-on and plasma-off conditions :

$$\frac{(\Gamma_{O_2}\gamma_{O_2} + \Gamma_O\gamma_O) \cdot K_{des}}{\Gamma_{O_2}\gamma_{O_2} \cdot K_{des}} = \frac{(8.732 \pm 0.013) \times 10^{-4}}{(5.108 \pm 0.009) \times 10^{-4}} = 1.71 \pm 0.04$$

$$\frac{\Gamma_O\gamma_O}{\Gamma_{O_2}\gamma_{O_2}} \approx 0.71$$

Direct oxygen radical measurements (Supporting Information, Figure S6) give the ratio $\frac{\Gamma_O}{\Gamma_{O_2}} = (2.91 \pm 1.58) \times 10^{-3}$ at this condition. The large relative uncertainty (54%) reflects the sensitivity of radical measurements at lower plasma power and propagates directly into the extracted sticking coefficient ratio. We calculate $\frac{\gamma_O}{\gamma_{O_2}} = 244$ ($\pm$133) under this experimental conditions, which is consistent within uncertainty with $\frac{\gamma_O}{\gamma_{O_2}} = 286$ ($\pm$110) obtained at U_{plasma} -1100 V (P_{plasma} 471 W), confirming that the sticking coefficient ratio is independent of plasma power over the range tested and consistent with literature values.

For completeness, a full comparison of the fitted products across both plasma power conditions is summarized here. Under plasma-off conditions, the fitted product $\Gamma_{O_2}\gamma_{O_2} \cdot K_{des}$ is (4.882 $\pm$ 0.007) $\times$ 10^{-4} s^{-2} at 471 W and (5.108 $\pm$ 0.009) $\times$ 10^{-4} s^{-2} at 341 W, a difference of 4.6%, consistent with the expected power-independence of K_{des} at fixed SOEC temperature. Under plasma-on conditions, the fitted product $(\Gamma_{O_2}\gamma_{O_2} + \Gamma_O\gamma_O) \cdot K_{des}$ is (1.395 $\pm$ 0.009) $\times$ 10^{-3} s^{-2} at 471 W and (8.732 $\pm$ 0.013) $\times$ 10^{-4} s^{-2} at 341 W, reflecting the increase in atomic oxygen flux with plasma power. The extracted sticking coefficient ratios $\frac{\gamma_O}{\gamma_{O_2}} = 286 \pm 110$ at 471 W and 244 ± 133 at 341 W are consistent within uncertainty across both conditions, confirming that $\frac{\gamma_O}{\gamma_{O_2}}$ is an intrinsic surface property independent of plasma power, and that the surface reaction model yields physically consistent parameters across the range of discharge conditions tested.

S4. Plasma radical and ion fluxes measurements

The radical probe (Figure S5) determines atomic oxygen density from the catalytic recombination heat deposited on a silver-coated thermocouple surface relative to an inert Al_2O_3-coated reference thermocouple; the temperature difference between the two surfaces is used to extract the oxygen radical flux. Temperature readings from each thermocouple were recorded using a NI 865662-01 Temperature Measurement Bundle and a cDAQ-T1100 data logger.

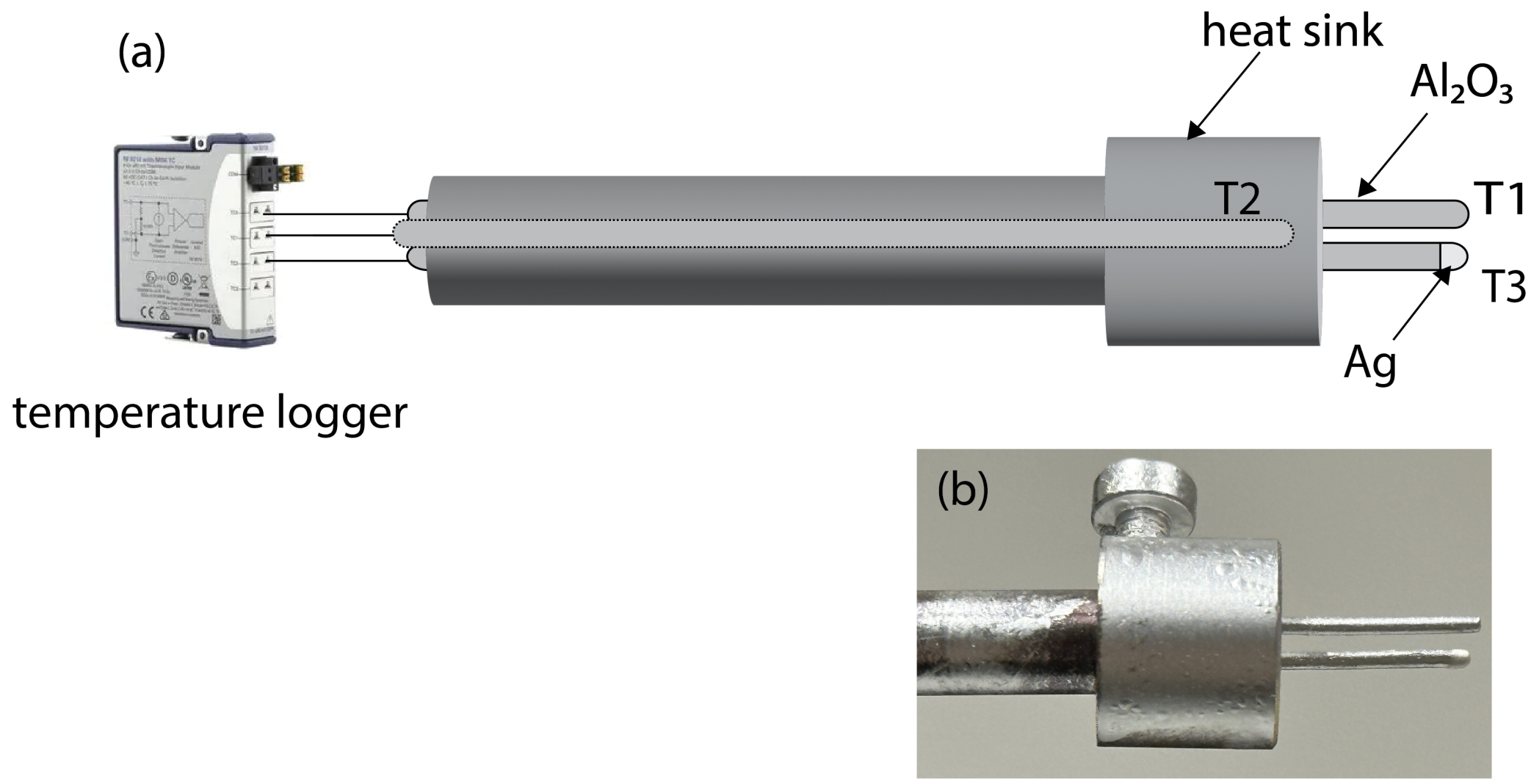

Figure S5: Radical Probe to measure the oxygen radical concentration. Adapted from the three-thermocouple probe design for nitrogen radical measurement.[6]

The cylindrical Langmuir probe was fabricated in-house from an iridium wire (diameter 0.25 mm, length 5 mm) welded onto an insulated tungsten support. Iridium was selected for its oxidation resistance. The probe was cleaned in pure helium plasma prior to each He-O_2 measurement. Its position was adjustable within the afterglow region adjacent to the SOEC surface. The probe was biased using a potentiostat (IviumStat.XRe, voltage range $\pm$50 V) swept from -50 V to slightly above the plasma potential, which ranged from -35 V to -21 V over the plasma power range of 200–500 W. All

probe measurements were conducted with the SOEC at room temperature (20 °C).

In the He-O_2 plasma, the measured averaged ion/electron fluxes ($\Gamma_e/\Gamma_{O_2^+}$) ranges from 2.1×10^{18} to 1.3×10^{19} m$^{-2}\cdot$s^{-1} over the plasma power range of 215 to 500 W (Figure S6). These fluxes are two to three orders of magnitude lower than Γ_O and five orders of magnitude lower than Γ_{O_2}, confirming that ion and electron contributions to surface oxygen coverage are negligible relative to neutral species under these conditions.

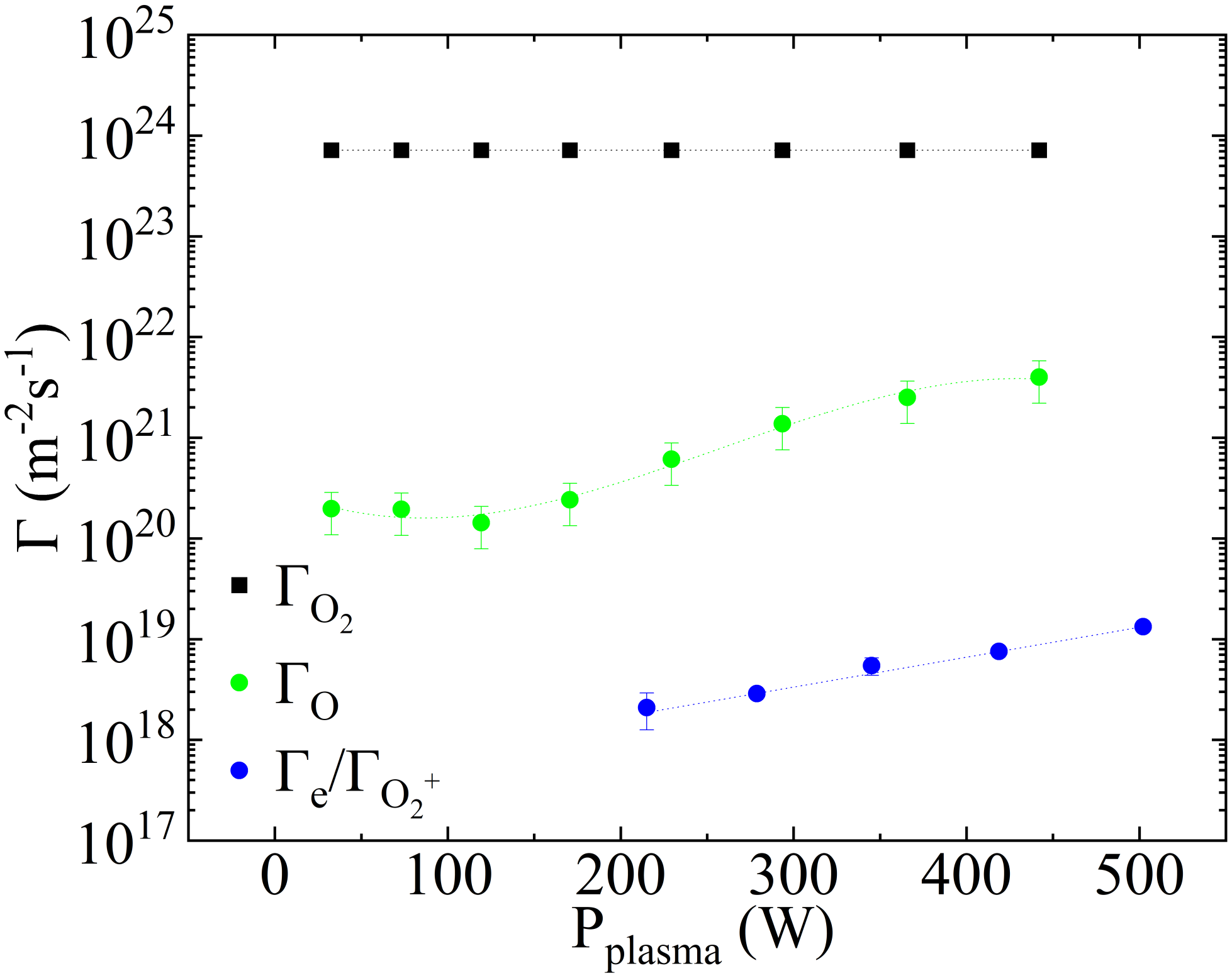

Figure S6: Flux measurements for 1 Torr He-O_2 plasma in the afterglow region, pO_2 0.2 Torr, inlet flow is 10 sccm.

References

[1] Takeda, Y.; Kanno, R.; Noda, M.; Tomida, Y.; Yamamoto, O. Cathodic Polarization Phenomena of Perovskite Oxide Electrodes with Stabilized Zirconia. *J. Electrochem. Soc.* **1987**, *134*, 2656–2661.

[2] van Heuveln, F. H.; Bouwmeester, H. J. M. Electrode Properties of Sr-Doped $LaMnO_3$ on Yttria-Stabilized Zirconia: II. Electrode Kinetics. *J. Electrochem. Soc.* **1997**, *144*, 134–140.

[3] Lieberman, M. A.; Lichtenberg, A. J. *Principles of Plasma Discharges and Materials Processing*; Wiley, 2005; pp 285–325.

[4] King, D. A.; Wells, M. G. Molecular Beam Investigation of Adsorption Kinetics on Bulk Metal Targets: Nitrogen on Tungsten. *Surf. Sci.* **1972**, *29*, 454–482.

[5] De Souza, R. A.; Kilner, J. A. Oxygen Transport in $La_{1-x}Sr_xMn_{1-y}Co_yO_{3\pm\delta}$ Perovskites: Part I. Oxygen Tracer Diffusion. *Solid State Ion.* **1998**, *106*, 175–187.

[6] Herrmann, A.; Krebaum, P. M.; Bera, S.; Tsampas, M. N.; van de Sanden, M. C. M. Enhanced Catalytic Probe Design for Mapping Radical Density in the Plasma Afterglow. *J. Phys. Chem. A* **2024**, *128*, 10080–10086.
